# Divergent responses of butterflies and bees to burning and grazing management in tallgrass prairies

**DOI:** 10.1002/ece3.9532

**Published:** 2022-12-04

**Authors:** Julia B. Leone, Nora P. Pennarola, Jennifer L. Larson, Karen Oberhauser, Diane L. Larson

**Affiliations:** ^1^ Department of Fisheries, Wildlife and Conservation Biology University of Minnesota St. Paul Minnesota USA; ^2^ Department of Entomology University of Minnesota St. Paul Minnesota USA; ^3^ U.S. Geological Survey, Northern Prairie Wildlife Research Center St. Paul Minnesota USA; ^4^ University of Wisconsin Arboretum, University of Wisconsin–Madison Madison Wisconsin USA

**Keywords:** grassland insect conservation, grassland management, ground‐nesting bees, pollinator conservation, prairie butterflies, prescribed fire

## Abstract

Butterflies and bees contribute significantly to grassland biodiversity and play important roles as pollinators and herbivores. Grassland conservation and management must be seen through the lens of insect conservation and management if these species are to thrive. In North America, grasslands are a product of climate and natural disturbances such as fire and grazing. These natural disturbances have changed considerably since European colonization and subsequent landscape fragmentation. The aim of this study was to better understand the impacts of fire and grazing management on butterfly and bee communities in tallgrass prairie, enabling land managers and conservationists to better protect and manage remnant prairie. We examined butterfly and bee abundance, species richness, and diversity in Minnesota tallgrass prairies managed by grazing or fire. In 2016 and 2017, we surveyed butterflies, bees, vegetation, and surrounding land use at 20 remnant prairies (10 burned and 10 grazed) with known management histories. Butterfly and bee abundance at our study sites were significantly negatively correlated. Butterfly abundance, but not species richness, was higher in burned than grazed prairies, and prairie‐associated grass‐feeding butterflies were more abundant at sites with higher plant species richness. Bee abundance was unrelated to management type but was higher at sites with sandier soils; bee species richness was positively associated with forb frequency. These findings highlight the challenges of designing management plans tailored to wide groups of pollinators and the potential pitfalls of using one group of pollinators as indicators for another. They also point to the importance of a mosaic of management practices across the prairie landscape.

## Graphical abstract

The aim of this study was to better understand the impacts of fire and grazing management on butterfly and bee communities in tallgrass prairie, enabling land managers and conservationists to better protect and manage remnant prairie. Butterfly and bee abundance at our study sites were significantly negatively correlated. Butterfly abundance was associated with management type, while bees were associated with forbs and soil type.

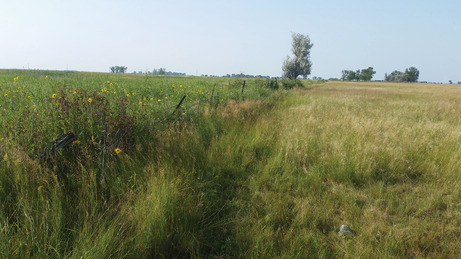

## 1 INTRODUCTION

Thoughtful and informed land management is necessary if declining native grasslands and their inhabitants are to persist. Butterflies and bees contribute significantly to grassland biodiversity and play important roles in ecosystem functioning. Butterfly adults are incidental pollinators, butterfly larvae are important herbivores (Scoble, 1992), and all life stages serve as food sources for birds and other animals. Bees are considered the most important pollinators both globally and in tallgrass prairie (Grimaldi, 1999). Worldwide declines in insect diversity and abundance are increasingly well‐documented (e.g., Biesmeijer et al., 2006; Cameron et al., 2011; Wagner, 2020), including the butterflies and bees that are the subjects of this study. Prairie specialist butterflies are declining in Iowa, Wisconsin, Minnesota, and Illinois tallgrass prairies (e.g., Schlicht et al., 2009; Swengel & Swengel, 2013; Swengel et al., 2011), and 10 of the 15 endangered, threatened, or special concern butterfly species in Minnesota are associated with tallgrass prairie (Minnesota Department of Natural Resources, 2013). Some of these, including the threatened Dakota Skipper (*Hesperia dacotae*) and federally endangered Poweshiek Skipperling (*Oarisma poweshiek*), were once among the most common butterflies in tallgrass prairie (Dana, 1991; Schlicht & Orwig, 1998). The federally endangered rusty‐patched bumble bee (*Bombus affinis*), which occurs in Minnesota, was abundant only twenty years ago and is now rarely found across most of its historic range (USFWS, 2016). The imperiled status of these insects warns us that common species are not resistant to declines faced by insects as a whole. Extensive changes to natural disturbance regimes in the Minnesota tallgrass prairie, coupled with habitat loss and fragmentation, are potential drivers of declines of once ubiquitous insect species. It is therefore increasingly important that grassland conservation and management take insects into consideration when developing management plans.

North American prairie evolved and was maintained for tens of thousands of years through ungulate grazing, lightning‐ignited fires, and indigenous fire management (Anderson, 2006; Middleton, 2013), which reduced woody plant growth. Land managers often attempt to mimic natural fire and grazing disturbances through prescribed fire and cattle grazing management (Brudvig et al., 2007). However, with so much of the historic extent of prairie gone and what remains scattered across a fragmented landscape, managers face increasing challenges when seeking to maintain remnant prairie (prairie that has never been plowed or converted to agriculture). At least 98% of Minnesota's approx. 7,285,000 hectares of the tallgrass prairie has been converted to agriculture or otherwise lost, and other tallgrass prairie states have suffered similar losses (Samson et al., 2004). This habitat loss and fragmentation results in substantial threats to biodiversity (e.g., Brudvig et al., 2015; Fahrig, 2003; Haddad et al., 2015; Summerville & Crist, 2001).

Although fire and grazing occurred concurrently or in response to one another historically (Anderson, 2006), managers today are often faced with the choice of either burning or grazing based on logistic (e.g., having the infrastructure to manage cattle or sufficient distance from human habitation to apply fire) or economic (e.g., willing livestock owners to graze on the remnant prairie or available trained personnel to apply fire) feasibility. Fire management has become more challenging as prairie remnants become fragmented, smaller, and more isolated. Managers are often constrained by the increased presence of humans, farmland, and roadways in the landscape because they must account for wind direction and smoke and the risk of fire escaping (Toledo et al., 2013). Additionally, leaving unburned refugia for prairie obligate insects (Swengel et al., 2011) becomes more difficult in smaller remnants. Although spatially dependent, these constraints can result in fire frequencies that are lower than many resource managers would consider optimal, and also lower than are used in most research studies on fire effects (e.g., Collins & Calabrese, 2012; Dickson et al., 2019). Management must respond to local conditions, and Midwestern tallgrass prairies rarely, if ever, receive the frequent fire that is more typical in places like Konza Prairie, where much of the influential research on fire and grazing originated.

Conservation grazing, in which domestic herbivores are used to further conservation goals (Asensio & Lauenroth, 2012), is one way to reduce potential threats of fire. However, today's conservation grazing is done almost exclusively with domesticated cattle, which preferentially graze different vegetation, prefer wetter areas, and move with different herd patterns than bison (Allred et al., 2011; Kohl et al., 2013; Plumb & Dodd, 1993). Grazing also requires partnerships with livestock owners who support conservation outcomes, and the additional fencing and water infrastructure required often makes grazing impractical. In addition to logistical challenges, it is not always clear which management strategy will produce the desired ecological outcomes. Grasslands are disturbance‐dependent landscapes, but there remains much debate about how best to practice disturbance management in the current landscape, especially with regard to insect conservation (e.g., Buckles & Harmon‐Threatt, 2019; Henderson et al., 2018).

Studies examining the impacts of fire and grazing management on butterflies and bees often find inconsistent results. Panzer (2002) and Thom et al. (2015) report that prairie remnant‐dependent butterfly species that overwinter above ground as eggs, larvae, or pupae are particularly vulnerable to fire, especially if there are few nearby refugia from which butterflies may recolonize a burned site (Driscoll et al., 2010; Swengel & Swengel, 2007). Swengel (1998) found that, in general, the majority of butterfly species studied occurred in greater abundance under mowing and grazing management than under rotational‐burning management. On the other hand, butterflies typically absent during the time when fires are set, such as monarchs (*Danaus plexippus*) (Leone et al., 2019; Moranz et al., 2012) and other migratory species, or that are in life stages that occur underground (e.g., *Maculinea* spp in Europe (Nowicki et al., 2015)) may not suffer negative effects of burning but instead benefit from habitat improvement. Vogel et al. (2007) found that while butterfly species richness did not differ between management practices, butterfly diversity indices were highest in burn‐only sites and species composition differed by management. In comparison, bees' responses to fire or grazing are influenced by their life history, including nesting location. Those that nest 10 cm or deeper underground (75% of ground‐nesting taxa) tolerate most grassland fires, which typically do not raise soil temperatures to lethal levels nor for lethal durations (Cane & Neff, 2011; DeBano et al., 1998). Fires can be more dangerous for insects that nest aboveground due to both nest combustion and lethal temperatures (Tooker & Hanks, 2004). Results have been mixed regarding the impact of grazing on grassland bees. Kimoto et al. (2012) found that grazing intensity had no significant effect on total bee abundance or species richness in the central Oregon prairie. There were differences in response between genera, with greater intensity grazing more negatively impacting *Bombus* (bumble bee) than *Lasioglossum* (sweat bee) abundance. Increased grazing intensity was also associated with a lower Shannon diversity in bees in the early season, potentially due to declines in floral resources. However, Carvell (2001) found a greater abundance of bumble bees in pastures grazed by cattle within the past year.

Butterflies are sometimes used as pollinator “indicator” taxa in ecological studies (Thomas, 2005), due to the comparative ease of sampling and identifying butterflies compared with bees. However, there is debate about their usefulness as indicators. Davis et al. (2008) found that butterfly and bee diversity were negatively correlated in Iowa tallgrass prairies, although management practices were not considered in their study. Management plans that assume similar responses from different pollinator groups may only benefit some species, while others are left out. It is essential for grassland management and butterfly and bee conservation that these assumptions are tested.

To inform better management of tallgrass prairie butterflies and bees, we investigated how bees and butterflies respond after ≥11 years of fire or grazing management as practiced by resource managers. We thus are considering the cumulative effects over time of these management practices on bees and butterflies, rather than the direct and immediate effects of fire or grazing on the organisms. Our goals were to assess (1) the effects of conservation grazing versus prescribed fire management on butterfly and bee abundance and richness and (2) whether butterflies and bees differ in their responses to fire versus grazing management. Specifically, we investigated the abundance and species richness of all observed butterflies and bees, as well as subsets of each: resource‐user butterflies, which represent observed butterflies seen using resources within managed sites, as opposed to flying through; prairie‐associated grass‐feeding butterflies, which we were interested in because of their relation to species of conservation concern; and soil‐excavating ground‐nesting bees, which are among the most abundant and speciose bee taxa typically collected in bee bowls.

While site management is important in shaping prairie bee and butterfly communities, it does not occur in isolation. We hypothesized that both butterfly and bee communities would be affected by management practices, but that their responses to fire vs grazing would differ and be mediated by local and landscape factors such as patch size, prairie habitat availability in the landscape, floral and host plant resources, and soil texture. Habitat patch size and the amount of suitable habitat in the surrounding landscape are known to positively impact bee and butterfly communities (Denning & Foster, 2018; Robinson et al., 2014; Topp et al., 2021). We expected these to be positively associated with the abundance or diversity of butterflies and bees. Forbs provide nectar for bees and butterflies, and pollen for bees (Denning & Foster, 2018; Öckinger & Smith, 2006; Winfree et al., 2011). We thus expected the abundance of butterflies and bees to be positively associated with forb frequency. Host plant resource availability is important in shaping butterfly communities (Dennis et al., 2011). Nine of Minnesota's endangered, threatened, and at‐risk butterfly species feed on native graminoids, as do all other members of the subfamily Hesperiinae (Hesperiidae) (Narem & Meyer, 2017; Scott, 1986). We expected butterfly and bee diversity to be positively associated with plant species richness and prairie‐associated grass‐feeding butterfly diversity to be positively associated with native graminoid frequency. For ground‐nesting bees, soil accessibility (i.e., bare soil) and texture are vitally important. Fire initially increases bare soil exposure, which can provide ground‐nesting bees with more nesting opportunities. Grazing also increases the amount of bare soil in grasslands, with higher intensities resulting in more bare ground (Kimoto et al., 2012). Despite the increase in bare soil exposure, we also expected ground‐nesting bee abundance to be negatively associated with grazing frequency and intensity, as cows can lead to increased soil compaction and inundation of soils (Alaoui et al., 2018; Batey, 2009; Buckles & Harmon‐Threatt, 2019).

Because the effects of land management can take years to appear, and because we wanted to provide insights directly relevant to the types of prairies with which land managers work in Minnesota, we chose study sites that were managed at least once during the eleven years prior to this study by state, federal, and private land managers and that were exclusively burned or grazed for at least eleven years prior to the beginning of our study.

## 2 METHODS

### 2.1 Study sites

**FIGURE 1:**
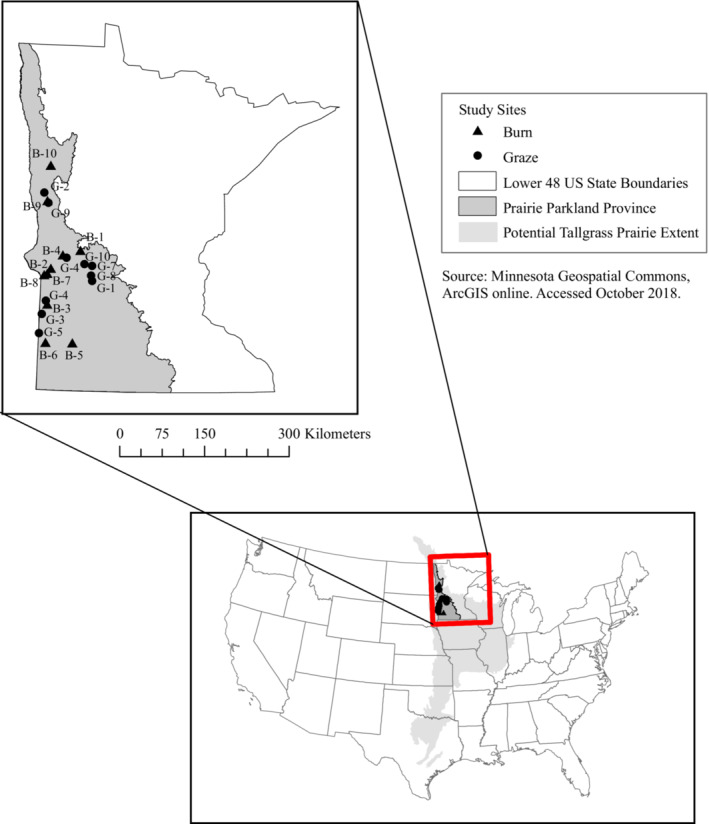
Map of burned (B 1–10; triangles) and grazed (G 1–10; circles) tallgrass prairie study sites within the prairie parkland province in Minnesota.

We chose 20 remnant, tallgrass prairie sites within the prairie parkland province in Minnesota (Figure 1) from candidate sites that had all been exclusively either burned or grazed by cattle between 2005 and 2015 (10 burned, 10 grazed). Sites represented a range of sizes (1.13–144.7 ha), prairie habitat in the surrounding landscape (0.15%–68%), years managed (1–13 years), time since fire (2–9 years), and cattle stocking rates (0.17–2.9 AUM, Animal Unit Month) (Appendix 1, Table A1). Management records, permits, and permissions were granted by owners (the US Fish and Wildlife Service, Minnesota Department of Natural Resources (MN DNR), The Nature Conservancy, and private landowners).

We created a 1.5‐km buffer (Greenleaf et al., 2007; Lane et al., 2020) around each site and calculated the percent of the prairie surrounding each site, not including the site itself, using ArcMap (v 10.5.1); see full methods in Larson et al. (2018). Briefly, we calculated the percent of land classified as prairie within the CropScape cropland data layer (Han et al., 2014), MN DNR native prairie and Reinvest in Minnesota‐MN Geospatial data (MN DNR), and South Dakota State University potentially undisturbed land (Bauman et al., 2016) within the 1.5‐km buffer around each site.

### 2.2 Sampling methods

Butterflies and bees were surveyed at sites three times in both 2016 (June 15 to August 31) and 2017 (May 14 to August 18), for a total of 117 surveys. One grazed site, (G‐1) was only surveyed in 2017. To address phenology differences across the north‐south range of sites, we surveyed sites from south to north. We conducted 60 bee and butterfly surveys at burned sites and 57 at grazed sites during the study.

To minimize the effect of time of day on sampling, sites surveyed in the afternoon during one visit were surveyed in the morning during the next visit and vice versa. To reduce weather‐related sampling variability, insect surveys were conducted between 09:30 h and 18:30 h (with 2 exceptions when surveying finished between 18:30 h and 19:00 h), when temperatures were above 20°C, sustained winds were less than 20 km/h, and cloud cover was <70% (15 exceptions) with no precipitation (Moranz et al., 2014; Pollard & Yates, 1993). Using available soil drainage data, we delineated wet, mesic, and/or dry prairie polygons for each site. Transects were delineated within each prairie‐type polygon prior to field sampling, and oriented parallel to elevation gradients. The total length of insect transects was the same at all sites: Butterfly transects were 400‐m long and bee transects were 180‐m long, sharing the same beginning points. For insect transect survey purposes, we sampled each prairie type in proportion to its portion of the total site area. For example, if a site was delineated as 50% mesic prairie, 40% wet prairie, and 10% dry prairie, we conducted 200 m of butterfly transects surveys along transects in the mesic prairie, 160 m in wet prairie, and 40 m in dry prairie. If a site was 20% mesic prairie and 80% wet prairie, we would conduct 80 m of butterfly transect surveys in mesic prairie and 320 m in wet prairie. We similarly distributed bee bowls proportionally along wet, mesic, and/or dry prairie transects. At some sites, one continuous transect did not fit and transects were broken into smaller sections due to prairie type, shape, or size; at these sites, transects were at least 20‐m apart to avoid sampling redundancy.

### 2.3 Butterfly surveys and identification

All butterfly surveys were conducted by the same observer using two methods. First, we used a modification of the standardized Pollard Walk for relative abundance (e.g., Pollard, 1977; Pollard & Yates, 1993), during which we walked transects at a steady pace of 10 m/minute and recorded each individual butterfly seen within a 5‐m imaginary box in front of the observer: 2.5 m on each side, 5 m ahead, and up to 5 m above the ground. This method provides relative abundance data and is used in the analyses that follow. The second method was a meandering walk, in which we conducted a time‐constrained walk of the site during each visit and recorded additional species not encountered during the Pollard Walk (individuals per species were not recorded). The length of the meandering walk is scaled with site size, lasting between 30 min and 2 h, and the timer was stopped while processing butterflies. Data from meandering walks were only used to assess species richness. Butterflies were sampled by sight identification, netted for identification and released, or collected for laboratory identification. Collected specimens were placed in individual glassine envelopes, labeled, placed in ethyl acetate jars while in the field, and transferred into a freezer until preparation. Species identifications were confirmed using Schlicht et al. (2007) and Opler and Malikul (1998). Collected voucher specimens are housed in the University of Minnesota Insect Collection. A list of all butterfly species observed is provided in Appendix 2, Table A2.

### 2.4 Bee surveys and identification

During each site visit, bees were surveyed in two ways, passively via pan traps (“bee bowls”), and actively, via netting, to achieve the most complete account of species at the sites.

We used 3.25 oz. plastic bowls in three colors (white, yellow, and blue) along 180 m of the same transects used for butterfly surveys, starting at their beginning point. We placed bee bowls after Pollard walks had taken place, to avoid flushing butterflies before they could be observed. The bowls were elevated on bamboo poles ~0.5 m above ground level. At 20‐m intervals, we placed one bowl on the transect and two additional bowls perpendicular to the transect, 5 m from the center bowl. Thirty bowls in total were placed at each site. This adaptation of the standardized bee bowl transect was made to create gaps in the transect through which cattle could pass without disturbing traps while maintaining a minimum distance of 5 m between bowls (Droege et al., 2010, 2017). We divided bee bowls proportionally between prairie types, such that the number of sets of traps on transects in each prairie type was proportional to that prairie type's contribution to the site. The bowls were filled with soapy water (water and Dawn© unscented dish soap) and left in place for approximately 24 h. Due to fieldwork logistical constraints, the time over which bowls were deployed varied from 1190 min (19.83 h) to 1670 min (27.83 h), with a median of 1415 min (23.58 h). All captured insects from a transect were placed in a single Whirl‐Pak bag and kept in a freezer until processed and pinned. Bee bowls were not placed at Site B‐5 during the second two visits in 2016 to avoid disrupting ongoing surveys by the MN DNR. Through a data‐sharing agreement, we obtained bee collection data from two DNR visits that occurred during this period of 2016. Three samples out of 117 were unusable; two were lost and one was unlabeled.

All site visits also included a time‐constrained meandering walk in which bees were netted when observed on flowers; the timer was stopped while processing bees. The length of the meandering walk is scaled with site size, lasting between 30 min and 2 h. Netted bees were placed individually in a glassine envelope, labeled with the date, time, and site name, and kept in an ethyl acetate jar until frozen for later processing. Data from this method were only used to supplement species richness data from bee bowls but not used in abundance analyses. All bee identification took place in the laboratory with the use of a stereomicroscope, using the following keys and guides: Gibbs (2010, 2011), Gibbs et al. (2013), LaBerge (1967, 1980), Laverty and Harder (1988), Ribble (1968), and Williams et al. (2014). Discover Life (Ascher & Pickering, 2018) was also consulted. A table of all bee species identified is included in Appendix 2, Table A3.

### 2.5 Vegetation surveys

Vegetation was sampled twice at each site, once in 2016 and once in 2017, in 0.5‐m × 2‐m plots along transects proportional in length to site size and prairie type (wet, mesic, dry); the number of plots was proportional to the size of the site, with a minimum of five and a maximum of 30. Transect length for plant surveys ranged from 36 to 1058 m and was dependent on the size and shape of the prairie‐type polygon within the site; distance between transects was at least 20 m. Starting and ending points of transects were a minimum of 10 m from site edges. The number of vegetation survey plots for each site (*n*), with a minimum plot number of 5 and asymptote of 30 was calculated using the following equation:

$$n=a\times \left(1–\exp\left(-b\times x\right)\right)$$

See Larson et al. (2020) for a complete description of how vegetation transects and plots were established.

Butterfly and bee transect surveys occurred along subsections of vegetation transects. Plant species richness, forb frequency, native graminoid frequency, and *Poa pratensis* and *Bromus inermis* (invasive graminoid) frequencies were calculated based on the presence of each detected species (number of occupied plots/total plots) (Appendix 1). We used plant frequency because sampling occurred throughout the growing season, so cover in early surveys would not be comparable to cover in later surveys (Elzinga et al., 1998). *Poa pratensis* and *Bromus inermis* are invasive thatch‐forming grasses that land managers seek to control through fire and grazing management. Five 10‐cm × 2.54‐cm soil cores were collected at each site along a randomly selected vegetation transect once in either 2016 or 2017, from which the proportion of sand was calculated (Appendix 1). Vegetation and soil methods are described fully in Larson et al. (2020).

## 3 ANALYSIS METHODS

### 3.1 Butterfly response variables

Four measures of butterfly abundance were modeled separately: total butterfly abundance, resource‐user abundance, non‐monarch butterfly abundance, and prairie‐associated grass‐feeding butterfly abundance. We summed total butterfly abundances from three survey visits at each site in 2016 and 2017 separately for each year, resulting in an index of butterfly relative abundance, which we hereafter refer to as total butterfly abundance (*n =* 39). We analyzed a subset of total abundance that included only butterflies that were observed using resources at a site (i.e., we removed butterflies from the analyses that had only been observed flying and not nectaring, basking, mating, ovipositing, or performing other activities related to site resource‐use), hereafter, “resource‐users.” We did this because butterflies only observed flying at a site may not be impacted by local management, especially for smaller sites and larger, more mobile species. We analyzed a subset of total abundance that included only non‐monarch butterflies. We did this because monarchs accounted for a large proportion of butterfly observations at our sites (Table A2) and we previously found them to be positively associated with fire at the same sites (Leone et al., 2019). We also analyzed prairie‐associated butterflies whose larvae feed on grasses based on Schlicht et al. (2007), Narem and Meyer (2017), and personal communications with local butterfly experts. These species, listed in Table A2, are of interest to us because many prairie‐associated butterflies in Minnesota and the Midwestern United States feed exclusively on native grasses in their larval stages, including the once‐common but now federally endangered *Oarisma poweshiek* and federally threatened *Hesperia dacotae*. The abundances of many species within the prairie‐associated group were too low to allow for species‐specific analyses, so we grouped all prairie‐associated grass‐feeding species together for analyses.

Butterfly species richness was estimated using the Chao 2 estimator (Chao, 1984; Colwell & Coddington, 1994). The suite of species observed at a site can be sensitive to bias due to the size of the site, the conditions during site visits, and the effort during surveys (Chao et al., 2014). Observed species richness can thus be an unreliable measure of the full community at a site, especially considering that some species are very rare and therefore unlikely to be detected. We calculated Chao 2 as:

$$S_{Y,T}+\frac{L_{Y,T}^{2}}{2M_{Y,T}}$$

The term *S*_*Y*,*T*_ represents the number of species observed during transect surveys plus meandering walk surveys at site *T* during year *Y*, *L*_*Y*,*T*_ is the number of species that occur in only one sample from site *T* during year *Y*, and *M*_*Y*,*T*_ is the number of species that occur in exactly two samples at site *T* during year *Y*. The estimated richness and the observed richness become more similar as the ratio of unique species to doubly observed species gets smaller. This is based upon the assumption that in the true community, many fewer species should occur in a single sample than in two samples. Thus, as the ratio of *L* to *M* gets smaller, the Chao 2 estimator approaches *S*. As species richness is a count of discrete species, a Poisson distribution is appropriate for models. We rounded the Chao 2 estimator to the nearest integer and used the fossil package (Vavrek, 2011) in R 3.6.2 (R Core Team, 2019) to calculate this estimator for each site in 2016 and 2017. Hereafter, “butterfly species richness” refers to the Chao 2 estimated value.

### 3.2 Bee response variables

Bee abundance was adjusted to account for the loss of bee bowls at grazed sites when cattle were present. The adjusted bee abundance was calculated as:

$$\left(\frac{\text{Total number of bees collected}}{\text{Total number of bowls retrieved}}\times 90\right)\;\text{rounded to the nearest integer}$$

This calculation estimates the number of bees that would have been collected had an entire set of traps (30 bowls × 3 visits = 90) been recovered in a given year. Rounding to the nearest integer allows for the use of the Poisson distribution, which is appropriate for count data. For most site visits, where all 30 bee bowls were recovered, the adjusted bee abundance and raw bee abundance were identical. Hereafter, “bee abundance” will refer to adjusted bee abundance.

Bee species richness was estimated using the Chao 2 estimator described above. Hereafter, “bee species richness” refers to the Chao 2 estimated value. We also analyzed a subset of total abundance and bee species richness that included only bees that excavate nests underground. Ground‐nesting bees were categorized according to an in‐progress database from Bartomeus et al. (2013).

### 3.3 Butterfly and bee models

The response variables described above were analyzed using Poisson distributed generalized linear mixed‐effects models (GLMMs). Predictor variables were selected *a priori* based on the literature and included management type as a categorical variable (burned, grazed), the percent of prairie within 1.5 km, site area, forb frequency, and the combined frequency of two invasive, thatch‐forming graminoids (*Poa pratensis* and *Bromus inermis*). Butterfly models included plant species richness and native graminoid frequency, to account for potential host plant associations. Bee models included the proportion of sand in the soils, as soil texture has important implications for ground‐nesting bees. We did not include the year as a fixed term because our study was not designed to test for differences between years. We used a two‐step modeling process for each response variable; we first built univariate models for each predictor variable, then built a global multivariate model including all predictor variables. Final models were selected for each response variable by using backward elimination to remove the least‐significant variables one at a time from the global multivariate model until the Akaike Information Criterion (AIC) value was not improved or all remaining predictor variables met a significance level of *p* < .05. If the model that best explained the response variable contained no variables significant at *p* < .05, we judged that the response could not be explained by any of the variables measured. Sites, and year nested within site, were included as random effects in all models. We tested the likelihood ratio between models with the random effects structure of year nested within site vs. models with just site as a random effect. We found that models that included year within site differed significantly from models that included only site as a random effect, indicating that these models can parameterize temporal variation despite the grouping factor having only two levels (Gomes, 2022). This method accounts for the well‐documented phenomenon of interannual variation in insect pollinators (e.g., Herrera, 1988; Price et al., 2005). We report the random intercept variance values for the final models in Appendix 3, Table A4.

We did not include additional management variables in our models because they were associated with management type (stocking rate was only relevant at grazed sites, time since fire only relevant at burned sites, and number of years managed not comparable between burned and grazed sites [Appendix 1, Table A1]). Instead, we built GLMMs with subsets of the data (burn‐only sites and graze‐only sites) to examine associations between all response variables and the predictor variables stocking rate and number of years managed at grazed sites and time since fire and number of years managed at burned sites.

We compared adjusted abundance and species richness for butterflies and bees using the Spearman's rank correlation and the function *cor.test* from the *stats* package in R (R Core Team, 2019).

For both butterflies and bees, analyses were conducted in R 3.6.2 (R Core Team, 2019) using the *glmer* function from the *lme4* package (Bates et al., 2015) and the *Anova* function, Type III sums of squares, from the *car* package (Fox & Weisberg, 2011).

We used nonmetric multidimensional scaling (NMS) with a Sorensen (Bray–Curtis) distance measure in PCOrd v. 7.08 (McCune & Mefford, 2018) to visualize butterfly and bee communities at burned and grazed study sites. We ran 250 permutations each of observed and randomized data. Community data were butterfly and bee species' abundance from butterfly transect walks and bee bowls; they did not include data from meandering walks. Years managed, proportion sand, and proportion clay were fitted as vectors on the graphs when *r* ^2^ ≥ .20. To help interpret the ordination, we obtained correlation coefficients of all butterfly and bee species with NMS axes.

## 4 RESULTS

### 4.1 Butterfly abundance

We observed 1239 butterflies during Pollard transect walks (625 in 2016 and 614 in 2017), 779 at sites managed with fire, and 460 at sites managed with grazing. Butterflies were observed at all study sites in both years.

**FIGURE 2:**
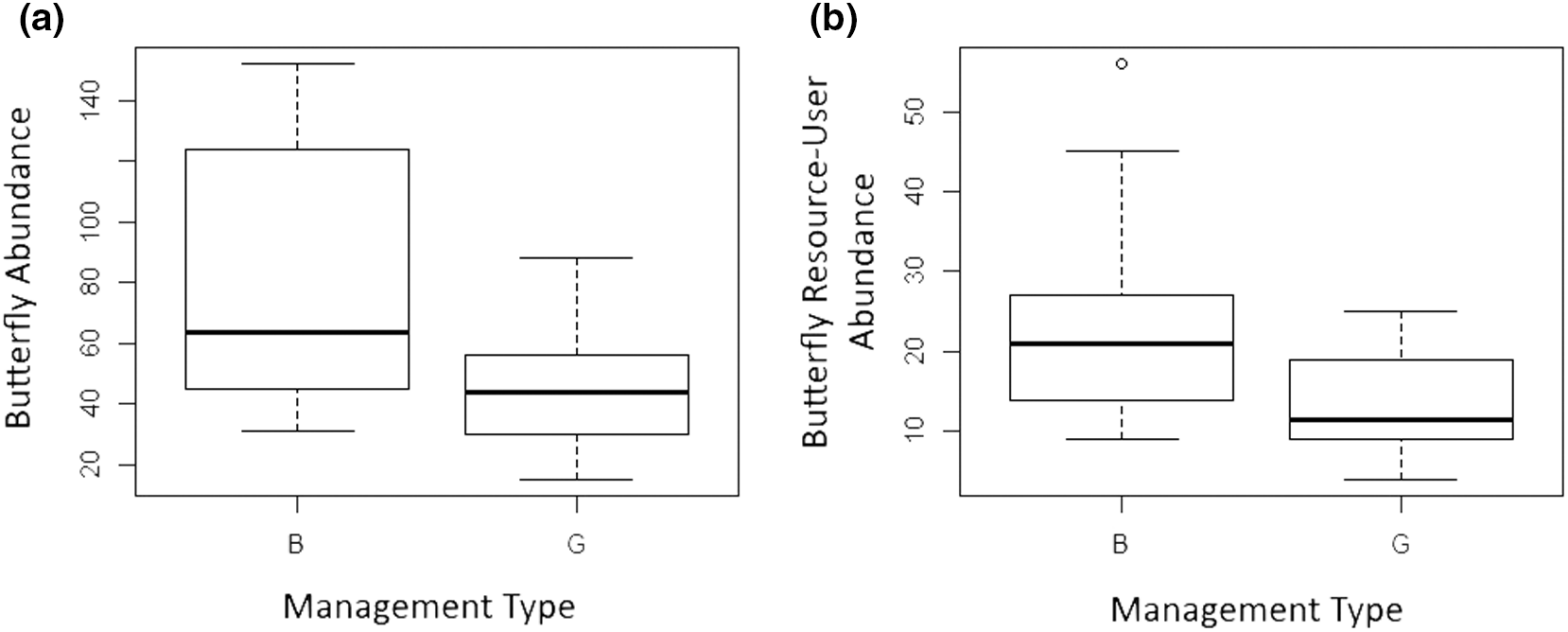
Total (a) and resource‐user (b) butterfly abundance at burned (B) and grazed (G) tallgrass prairie sites within the prairie parkland province in Minnesota, USA. Box plots depict the minimum, first quartile, median, third quartile, and maximum, with outliers depicted as single points.

Total butterfly abundance was close to two times higher at sites managed with fire than those managed with grazing (*z* = −2.332, *p* = .0197; Figure 2a); all other predictor variables were removed during backward elimination. The abundance of butterfly resource‐users and non‐monarch butterflies was also higher at burned sites than grazed sites (*z* = −2.22, *p* = .0264; Figure 2b and *z* = −0.4177, *p* = .0413, respectively), with management type as the only significant predictor variable after backward elimination in both cases. The abundance of prairie‐associated grass‐feeding butterflies was similar in burned and grazed sites (*z* = 0.069, *p* = .9448). The model with the lowest AIC value (ΔAIC > 2; Arnold, 2010) for prairie‐associated grass‐feeding butterfly abundance after backward elimination included only plant species richness (*z* = 1.680, *p* = .0929), which was positively, but not significantly, associated with abundance (alpha = 0.05). Other habitat variables did not explain any variation in the number of prairie‐associated grass‐feeding butterflies. We observed no prairie‐associated grass‐feeding butterflies in either 2016 or 2017 in four sites, two additional sites had no observations in 2016, and fewer than five individuals were observed at four of the occupied sites.

Total, resource‐user, non‐monarch, and prairie‐associated grass‐feeding butterfly abundance was similar at grazed sites with different stocking rates and numbers of years managed and at burned sites with different times since fire and number of years managed.

Univariate model results for all butterfly abundance response variables are presented in Appendix 4, Tables A5, A6, A7.

### 4.2 Butterfly species richness

We observed 39 butterfly species over the course of two summers; 36 in 2016 and 32 in 2017; 34 at sites managed with fire and 34 at sites managed with grazing (Table A2). Species composition differed somewhat between management types; five species were seen only at grazed sites (*Hesperia leonardus*, *Poanes viator*, *Thymelicus lineola*, *Coenonympha tullia*, and *Polites themistocles*) and four species were seen only at burned sites (*Echinargus isola*, *Satyrium acadica*, *Satyrium edwardsii*, and *Pyrgus communis*). Fewer than five individuals were observed for all species seen only at burned sites or only at grazed sites except for *Polites themistocles* (11) and *Pyrgus communis* (11). About one‐sixth (198) of observed butterflies were monarchs (*Danaus plexippus*), as previously described in Leone et al. (2019).

Butterfly species richness was similar at burned and grazed sites; no predictor variables were significant in the model. Butterfly species richness was similar at grazed sites with different stocking rates and numbers of years managed, and at burned sites with different times since fire and number of years managed. Univariate model results for butterfly species richness are presented in Appendix 4, Table A8.

### 4.3 Butterfly community composition

**FIGURE 3:**
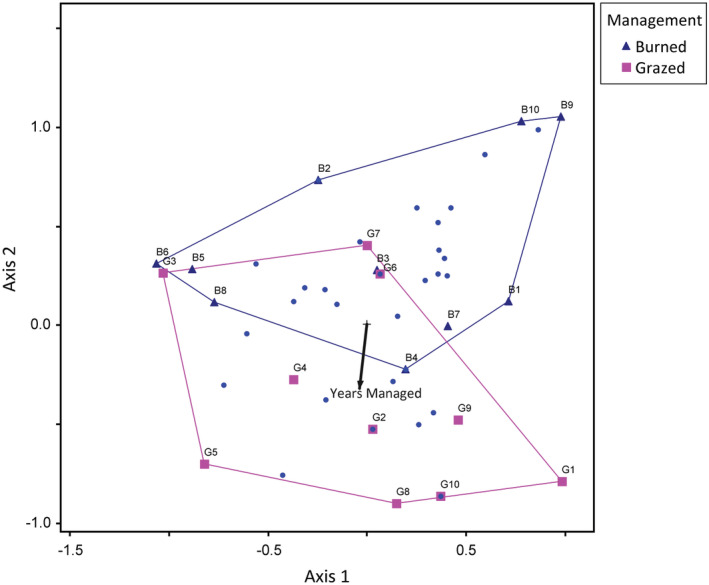
Sites within‐species space for nonmetric multidimensional scaling analysis of butterflies on grazed (pink squares) and burned (blue triangles) sites, axes 1 and 2. The second axis represented 28% of the variation in the data and was correlated with years managed (*r* = −.511). Vectors are proportional to the strength of the correlation with the axes. See Appendix 5, Table A13 for all correlations between butterfly species and NMS axes.

The first two axes in the butterfly NMS (stress = 9.5 with 45 iterations for a 3‐dimensional solution) indicated that butterfly communities in burned and grazed sites were quite distinct (Figure 3). The second axis represented 28% of the variation in the data and was correlated with years managed (*r* = −.511). The prairie‐associated grass feeders *Hesperia leonardus* (*r* = −.346), *Polites themistocles* (*r* = −.416), and *Coenonympha tullia* (*r* = −.346), as well as the skippers *Ancyloxypha numitor* (*r* = −.445) and *Poanes viator* (*r* = −.346) were most strongly positively associated with years managed. The prairie‐associated grass feeder *Cercyonis pegala* (*r* = .587), as well as *Colias* sp. (*r* = .418), *Danaus plexippus* (*r* = .583), *Phyciodes* sp. (*r* = .683), and *Speyeria cybele* (*r* = .584) were most strongly negatively associated with years managed (Appendix 5, Table A13).

### 4.4 Bee abundance

We collected 11,969 bees in bowl traps in the summers of 2016 and 2017. A univariate analysis of the effect of the duration of bee bowl deployment on adjusted bee abundance showed no significant correlation (*z* = 0.729, *p* = .4661).

**FIGURE 4:**
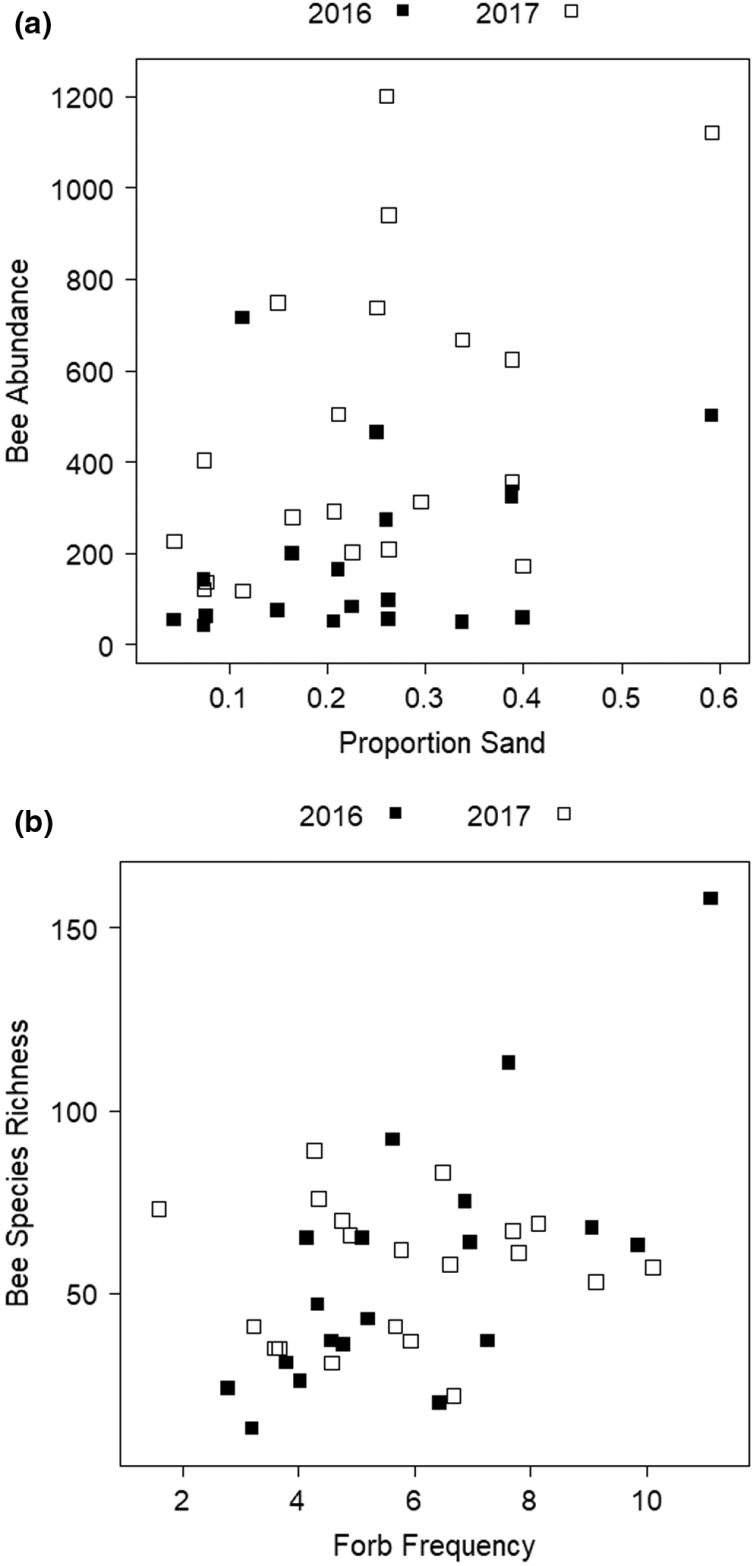
Relationship between (a) bee abundance and proportion sand and (b) bee species richness (Chao2) and forb frequency at sites in 2016 (black) and 2017 (white).

Total bee abundance was higher at sites with sandier soils (*z* = 2.421, *p* = .0155; Figure 4a); no other variables were significant in the final multivariate model. The abundance of soil‐excavating ground‐nesting bees was also higher at sites with sandier soils (*z* = 2.456, *p* = .014).

Neither time since fire nor the number of years managed with fire had a significant effect on total bee abundance or soil‐excavating ground‐nesting bee abundance, nor did stocking rate or the number of years managed with grazing. Univariate model results for bee abundance response variables are presented in Appendix 4, Tables A9, A10, A11, A12.

### 4.5 Bee species richness

We identified 119 species (30 genera) in our 2016 and 2017 collections. Sixty‐two specimens were not identified as species or species complex and were not included in richness analyses. One hundred two species were collected at burned sites, 25 of which were exclusive to burned sites, and 94 species at grazed sites, 17 of which were exclusive to grazed sites (Table A3). Twenty‐seven species were represented by only a single specimen (“singletons”), and 18 species were represented by two specimens (“doubletons”) (Table A3). Of the 119 species of bees we collected, 86 (72.2%) are soil‐excavating ground‐nesters and 11 (9.2%) occupy existing cavities (Table A3). Approximately 88% of individuals collected (11,004 of 12,540) are in the family Halictidae, bees that are mostly small ground‐nesters that generally prefer sandier soils (Cane, 1991; Potts & Willmer, 1997).

The final multivariate model for bee species richness included forb frequency, which was positively associated with species richness (*z* = 2.99, *p* = .0028; Figure 4b), and site area, which was negatively, but not significantly, correlated with species richness (*z* = −1.511, *p* = .1308).

None of the predictor variables tested were associated with ground‐nesting bee species richness.

Neither time since fire nor the number of years managed with fire had a significant effect on total bee species richness or soil‐excavating ground‐nesting bee species richness. The number of years managed with grazing had a significant effect on total bee species richness (*z* = −2.367, *p* = .018), with fewer bee species found at sites grazed more frequently. Neither the stocking rate nor the number of years managed with grazing had a significant effect on soil‐excavating ground‐nesting bee species richness.

Univariate model results for bee species richness are presented in Appendix 4.

### 4.6 Bee community composition

**FIGURE 5:**
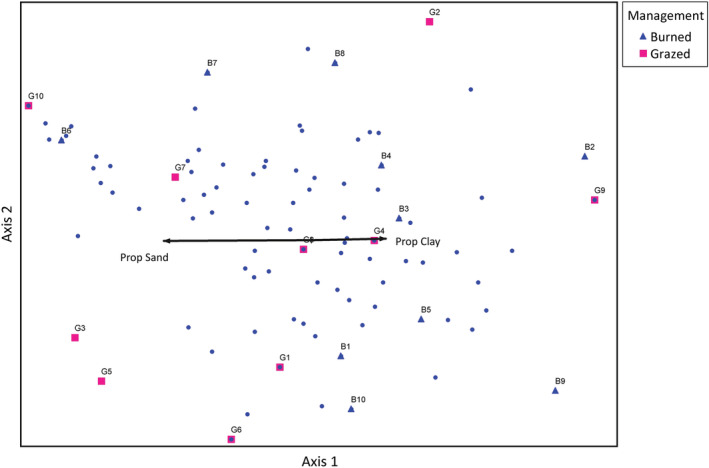
Sites within species space for nonmetric multidimensional scaling analysis of bees on grazed (pink squares) and burned (blue triangles) sites, axes 1 and 2. The first axis represented 52% of the variation in the data and was correlated with proportion sand (*r* = −.722) and proportion clay (*r* = .514). Vectors are proportional to the strength of the correlation with the axes. See Appendix 5, Table A14 for all correlations between bee species and NMS axes.

The first two axes in the NMS (stress = 7.4 with 78 iterations for a 3‐dimensional solution) indicated that bee communities overlap broadly between burned and grazed sites (Figure 5). The first axis represented 52% of the variation in the data and was correlated with the proportion sand (*r* = −.722) and proportion clay (*r* = .514). *Bombus vagans* (*r* = .540), *Hylaeus mesillae* (*r* = .569), *Lasioglossum ephialtum* (*r* = .633), and *Melissodes trinodis* (*r* = .457) were most strongly positively associated with proportion clay. *Agapostemon virescens* (*r* = −.646), *Bombus auricomus* (*r* = −.510), *Dianthidium simile* (*r* = −.541), *Eucera hamata* (*r* = −.568), *Halictus ligatus* (*r* = −.581), and *Lasioglossum pruinosum* (*r* = −.689) were most strongly positively associated with proportion sand (Appendix 5, Table A14).

### 4.7 Relationship between butterflies and bees

**FIGURE 6:**
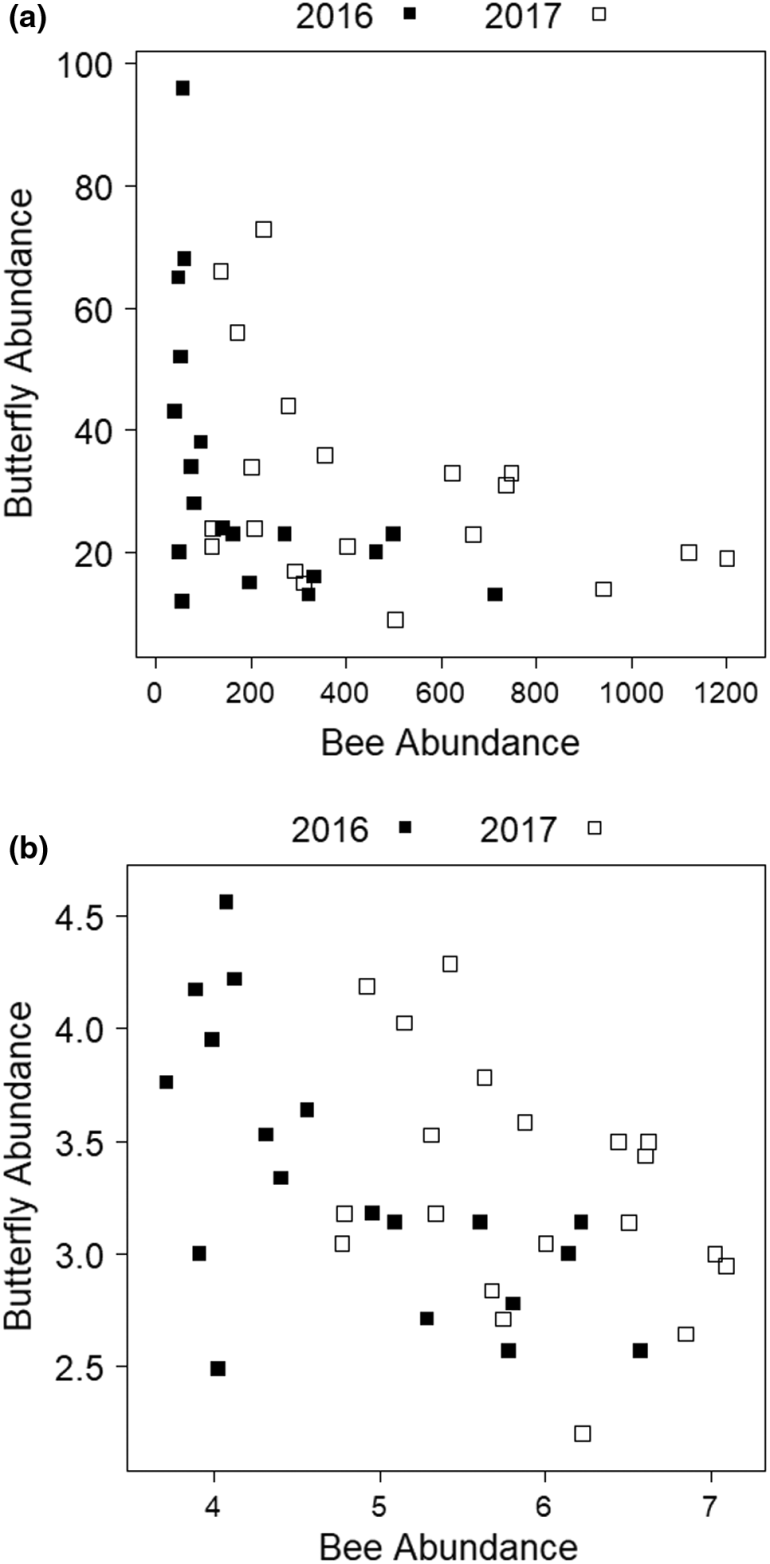
Relationship between bee abundance and butterfly abundance at sites in 2016 (black) and 2017 (white) in (a) linear scale and (b) log_10_ scale.

Butterfly and bee abundance at our study sites were significantly negatively correlated (*r*_s_ = −.48, *n* = 39, *p* = .0019; Figure 6). Butterfly and bee species richness were not correlated (*r*_s_ = .026, *n* = 39, *p* = .8745).

## 5 DISCUSSION

Butterfly abundance differed between burned and grazed remnant prairie, but bee abundance and species richness were related to sand and forb frequency at our study sites. These findings highlight the challenges of designing coherent management plans tailored to wide groups of pollinators and the dangers of using one group of pollinators as indicators for another (Table 1).

**TABLE 1:** Butterfly and bee responses to fire versus grazing in Minnesota tallgrass prairie: significant associations between response variables and predictor variables in final models after backward selection.

Response variable	Predictor variables			
	Management type	Plant species richness	Forb frequency	Proportion sand
Total butterfly abundance	0.0197**	ns	ns	ns
Butterfly resource‐user abundance	0.0264**	ns	ns	ns
Non‐monarch butterfly abundance	0.0413*	ns	ns	ns
Prairie‐associated grass‐feeding butterfly abundance	ns	0.0929	ns	ns
Bee abundance	ns	ns	ns	0.0155**
Ground‐nesting bee abundance	ns	ns	ns	0.014**
Bee species richness	ns	ns	0.0028***	ns

*Note*: Positive association with management type indicates that values were higher in burned (vs. grazed) sites and “ns” is not significant. Significance codes: 0.001 “***”; 0.01 “**”; 0.05 “*”.

We expected any butterfly response to management to be mediated by the influence of local or landscape vegetation. However, the vegetation variables we assessed (plant species richness, forb frequency, native graminoid frequency, frequency of invasive grasses *Poa pratensis* and *Bromus inermis*), site area, and the percent of prairie in the surrounding 1.5‐km buffer around each site were not correlated with butterfly abundance or species richness. This is in contrast to previous studies, which found positive associations between butterflies and site area and surrounding habitat (Kral et al., 2018; Robinson et al., 2014; Topp et al., 2021), nectar resources (Öckinger & Smith, 2006; Vogel et al., 2007; Winfree et al., 2011) and host plants (Dennis et al., 2011). The lack of associations we found with local and landscape variables may be partially explained by the fact that there was no clear separation between vegetation characteristics based on management type at our study sites (Larson et al., 2020; Leone et al., 2019). For example, plant communities were similar on burned and grazed sites (which included the sites in this study), although 28% of plant species occurred on only one or the other of the management types (Larson et al., 2020). Topp et al. (2021) found that fire was indirectly associated with butterfly abundance and species richness through its effect on the vegetation; no such indirect effects of management were documented at our sites.

Our finding that butterfly species richness did not differ based on management is consistent with others (Moranz et al., 2012; Vogel et al., 2007). However, our finding of higher butterfly abundance at sites managed with fire compared with grazing is more nuanced, and previous studies are more varied in their results. We note that fire frequencies at our sites (1–3 times in a 11‐year period) were sometimes much lower than in otherwise comparable studies. Vogel et al. (2007) found that most habitat generalists did not differ in abundance among management practices, although they reported a higher abundance of *D. plexippus* and *Colias eurytheme* in sites managed with only grazing compared to those managed with only burning; burn frequencies varied from 1–3 times in an 8‐year period. In comparison, we found similar *C. eurytheme* abundance between grazed and burned sites and nearly three times as many *D. plexippus* at burned compared with grazed sites (Table A2), which may be driving some of the patterns in overall abundance in our models (see Leone et al. (2019) for a more in‐depth analysis of *D. plexippus*). Our finding that *Speyeria idalia* abundance was higher at burned compared with grazed sites is supported by Vogel et al. (2007). Our findings are also consistent with Moranz et al. (2012), who reported the highest population densities of *C. pegala*, *S. idalia*, and *D. plexippus* in burn‐only treatments (Table A2). By contrast, Vogel et al., 2007 found that among habitat specialists, *Cercyonis pegala* abundance was higher in grazed than burned sites. Clearly, species identities influence butterfly responses to management.

In contrast to our results for total and resource‐user butterfly abundance, the abundance of prairie‐associated grass‐feeding butterflies did not differ between burned and grazed sites in our study. The positive relationship between the abundance of these butterflies and plant species richness combined with the fact that plant species richness did not differ between management types at our study sites (Larson et al., 2020) suggests that this association is unrelated to fire or grazing. Many grass‐feeding prairie‐associated butterfly species have seen precipitous declines in recent decades; in fact, many such species were not observed during this study (e.g., *Oarisma poweshiek*, *O. garita*, *Hesperia ottoe*, *H. dacotae*, and *H. uncas*) (Minnesota Department of Natural Resources, 2013; Schlicht et al., 2009; Swengel et al., 2011). The species in this group that we did observe were generally in low abundances. However, community composition and NMS results help differentiate species responses. Of the five species we included in the prairie‐associated grass‐feeding butterfly group, only *C. pegala*, the most abundant species in this group, was more abundant at burned sites than grazed sites (Table A2). Three of the remaining four species, *H. leonardus*, *P. themistocles*, and *C. tullia* were observed only at grazed sites (Table A2) and had strong positive associations in NMS with years managed (Figure 3; Appendix 5). Although many of these butterflies are included in studies of tallgrass prairie butterflies (e.g., Davis et al., 2008; Moranz et al., 2012; Schlicht et al., 2009; Swengel et al., 2011; Vogel et al., 2007), few studies have compared the impacts of management strategies for them (but see Swengel, 1998). Low abundances, specialized life histories, and association with plant species richness suggest that a more targeted study may be needed for these species of concern.

Differences in time since fire have been found to influence butterfly abundance. However, we found no effect of time since fire on butterfly abundance or on butterfly species richness in our study. This is in contrast to Vogel et al. (2010), who reported a positive association between butterfly abundance and time since fire, with 50‐ to 70‐month recovery times postfire in the Loess Hills of Iowa. Significant positive postburn responses to fire have also been documented for monarch butterflies and their milkweed host plants within one to two years following fire (e.g., Baum & Sharber, 2012; Rudolph et al., 2006). By contrast, lower butterfly abundance has been documented at burn‐only prairies than burn‐and‐graze prairies with a fire rotation of 2–6 years (Vogel et al., 2007). Because none of our sites were burned during the study or the preceding year (2015), differences in butterfly abundance are unlikely to reflect qualitative differences in nectar or host plant resources as a direct result of fire. Butterfly populations could have recovered from any negative impacts of fire at our study sites prior to surveying.

Another possible explanation for the higher abundance of butterflies at burned prairies compared with grazed prairies is a negative effect of grazing, rather than a positive association with fire. Our observation of fewer butterflies at grazed sites may indicate that grazing has a direct negative impact on butterflies through the consumption of eggs, larvae, or pupae. Leone et al. (2019) reported a negative correlation between monarch abundance and stocking rate at grazed sites; our data included the monarch data from Leone et al. (2019) and accounted for about one‐sixth of the total butterfly abundance reported in this study. Although the stocking rate and the number of years a site was grazed were not correlated with butterfly abundance in this study, there may be indirect effects of grazing that we did not quantify; we only measured the frequency and not the quality of plant species. Although forb frequency did not differ between our burned and grazed study sites (Larson et al., 2020), frequent fire has been shown to increase nectar availability (Rudolph et al., 2006); grazing may also reduce the amount of floral resources. We did not quantify floral resources but did observe cattle consuming flowers. Grazing reduces vegetation height, and several studies have found that butterflies prefer taller vegetation (Berg et al., 2013; Öckinger & Smith, 2006; Poyry et al., 2006).

Neither bee abundance nor species richness were influenced by management type in our study; this does not necessarily mean that bees do not respond to management but may mean that burning and grazing are functionally equivalent for prairie bee populations. Other studies (e.g., Buckles & Harmon‐Threatt, 2019; Griffin et al., 2021; Harmon‐Threatt & Chin, 2016) have found that management affects the structure of grassland bee communities. Our findings may be in part a result of how we treated management type, with burning and grazing as two distinct categories. Buckles and Harmon‐Threatt (2019), for example, compared burning, burning‐and‐haying, and patch‐burn‐grazing. Similarly, Griffin et al.'s (2021) web of restored prairie plantings was burned every one to three years, with bison grazing on a subset. It is also possible that the species most sensitive to disturbance type may have already fallen out of the prairie community, after more than a century of fragmentation, development, and agricultural intensification in the surrounding landscape. Comparisons to historical collections would be a worthy avenue for future research. The few bee species restricted to burned or grazed sites (Table A3) are only represented by one or two individuals, making any conclusions about their true exclusivity impossible. These species may be rarely occurring, or rarely captured using our techniques, making their detection at either management type just as unlikely.

Bees generally and the subset of soil‐excavating ground‐nesters were more abundant in sites with sandier soils. Different bee species prefer to nest in soils of different textures, although relatively few bees are associated with clay‐rich soils; most prefer sandy loams (Cane, 1991). These soils are easier to excavate and less susceptible to flooding than silt‐ or clay‐heavy soils (Skiba & Ball, 2002). Our bee community analyses also support the importance of soil texture in shaping the bee community; the proportions of sand and clay in soils were relatively strongly correlated with the first axis of the NMS, which explained most of the variation in the community (Figure 5). Analysis of soil‐excavating ground‐nesting bees, which represent the most abundantly collected bees in our samples, showed the sandiness of soils as the only significant predictor of their abundance. This indicates that soil‐excavating ground‐nesters are driving patterns of bee abundance. It may also indicate, as noted below, that biases in taxa collected by bee bowls are influencing analyses.

The response of the bee community to grazing is not a simple one. While soil‐excavating ground‐nesters have an important influence on models of total bee abundance, there are also signals of the importance of aboveground nesters and nonexcavators in grazed prairies. The frequency of grazing, measured as the number of years within the previous 10 years that a prairie was grazed, had a significant negative effect on total bee species richness. Like Kimoto et al. (2012), our best fit model did not include stocking rate as a significant predictor of bee abundance or species richness. Kimoto et al. (2012), offers us another point of comparison; they found that the abundance of the generally soil‐excavating genus *Lasioglossum* was less negatively impacted by grazing than the generally nonexcavating genus *Bombus*. Contrary to our expectations, there was a negative relationship between grazing frequency and species richness of the whole prairie bee community while the community of soil‐excavating ground‐nesters was not impacted in our study. While we expected that increased grazing frequency would compact soils, making bee nests more prone to inundation (Alaoui et al., 2018; Batey, 2009) and thus limiting soil‐excavating ground‐nesting bees' ability to make use of grazed sites (Buckles & Harmon‐Threatt, 2019), we detected nothing to indicate this.

Although the frequency of grazing had a significant effect on total bee species richness at grazed sites, forb frequency was the only significant predictor of bee species richness across all sites, a finding in line with prior research that documented floral resource availability as a limiting factor for bees (e.g., Inari et al., 2012; Ogilvie et al., 2017; Roulston & Goodell, 2011) and other pollinator communities (Sjödin, 2007). At our study sites, forb frequency itself was not significantly impacted by management type (Larson et al., 2020), but the lack of an association between management type and bee species richness was surprising, nonetheless. Fire may increase the length of the flowering season (Wrobleski & Kauffman, 2003), benefiting bees with relatively long flight periods, like bumble bees (Mola & Williams, 2018). While we did not document the flowering status of plants in our plots, the increased flowering season length documented by Mola and Williams (2018) was not explained by a shifting floral community; rather the same plant species seen at unburned sites flowered longer at burned sites.

We found that an 11‐year history of burning and grazing, in isolation, does not predict bee abundance or species richness. This equivalency of abundance and richness between burned and grazed prairies, as well as the lack of significant distinction between the communities making up burned and grazed prairies (Figure 5), may be the result of dispersal from other sites, indicating that there is resilience in isolated fragments. While we expected the amount of prairie in the surrounding landscape to have a significant positive effect on bee abundance and species richness as some studies have found (Eycott et al., 2012; Steffan‐Dewenter et al., 2002; Woodcock et al., 2013), we found no such relationship. This could instead support findings by Jauker et al. (2009) that the quality of the dominantly agricultural matrix in which semi‐natural grassland habitats exist has no significant effect on bee abundance. Importantly, the majority of individuals we collected do not rely solely upon prairie fragments; the four most abundant bee species in our study, *Lasioglossum pruinosum, L. albipenne*, *L. versatum*, and *Augochlorella aurata* (54% of all bees collected) are widely distributed across North America in various habitats (Coelho, 2004; Gibbs, 2011). Management effects, while potentially destructive to some individuals, may have minimal impacts on these species at a landscape scale, leading to seeming equivalency between management approaches for bee abundance and species richness. This is supported by our finding that management did not affect the abundance or presence of soil‐excavating ground‐nesters, which include the four species listed above.

Butterfly and bee abundance were negatively correlated, and we found no correlation between butterfly and bee species richness. Because associated predictor variables differed between butterflies and bees in our models, we urge caution in the use of one as an indicator of habitat suitability for the other. Davis et al. (2008) also found a negative correlation between bee and butterfly diversity in Iowa tallgrass prairie, citing potential competitive exclusion for nectar resources, or differences in resource preferences driving habitat selection. While we are unable to assess mechanisms driving habitat selection within the scope of this study, bees and butterflies have different requirements for reproduction, most notably appropriate nesting sites for bees and larval host plants for butterflies. Thus, while butterflies may be good indicators of change in some cases (Thomas, 2005), our study highlights their inadequacy as predictors of bee abundance and richness.

This retrospective study offered a duration of a single management type that would have been impossible to achieve through experimental manipulation in the time frame of this project. Additionally, the tallgrass prairie is a rare resource, and land managers are tasked with protecting and promoting that resource, whether for the public or for their herds. An observational study allowed us to work with land managers without compromising their missions; many of the managers worked with us in the hope that our findings could inform future management decisions on these very same lands. However, the retrospective nature of this study imposes some limitations. The lack of experimental manipulation made parsing out the direct and indirect effects of management difficult. We were also limited in our ability to control the extent of variation in factors unrelated to management, such as site area or latitude. Controls for variation in sites had to be made at the time of site selection, but it is possible that variables outside of our consideration, such as site history before 2005, could obscure signals. Interannual variation in insects, including among bees and butterflies, is well‐documented (e.g., Fishbein & Venable, 1996; Herrera, 1988; Price et al., 2005). We did our best to account for this background temporal variation by including year as a random effect in our models and reporting the random intercept variance in Appendix 3. However, we recognize the limitation imposed by two years of sampling data across highly variable populations.

Additionally, bee bowls are known to have limitations (e.g., Cane et al., 2000; Portman et al., 2020; Roulston et al., 2007). While bee bowls have been widely used in recent decades, they were not used historically, making comparisons with previous indices of prairie bee communities difficult (Portman et al., 2020 **)**. Bee bowl samples are biased towards certain taxonomic groups, with members of the family Halictidae over‐represented as compared to other collection means (e.g., Droege et al., 2010; Geroff et al., 2014; Griffin et al., 2017). Bees may also be drawn to bee bowls from the surrounding areas, especially when flowers are scarce (Kuhlman et al., 2021), making our samples a measure of both the surveyed site and the surrounding matrix of grassland, agriculture, and development (Baum & Wallen, 2011; Roulston et al., 2007). These last two points—the taxonomic bias and the potential attraction outside of the study area—may be driving results. The effect of sandy soils may be amplified by the fact that bee bowls attract the very bees that prefer sandy soils, obscuring other signals. The lack of significance of management type may be because bee bowl samples are drawing bees in from the wider area, where disturbance and habitats are more homogenized. However, we did attempt to curb these limitations by including a meandering walk to capture a wider breadth of bee species richness than found in bee bowls alone. Additionally, our analyses included the percent of prairie in the 1.5 km surrounding the sites, thus providing a measure of the broader habitat matrix that could account for unknown variation brought by bee bowls' attraction of bees from outside the study site. Ultimately, collection methods will always shape the sample of the community they provide. We present these limitations here in acknowledgment of that fact and encourage future studies to take them into account.

While fire and grazing both supply necessary disturbance to tallgrass prairie (Allred et al., 2011; Anderson, 2006; Carvell, 2001; Damhoureyeh & Hartnett, 1997; Harmon‐Threatt & Chin, 2016), they are not inherently exclusive processes. Historically, they would have co‐occurred across North America's grasslands, and many land management agencies have begun recoupling these processes. Patch‐burn grazing, in which cattle are set to graze on recently burned vegetation, is increasingly implemented to create a patchwork of heavily and lightly disturbed areas (Fuhlendorf et al., 2009; Helzer & Steuter, 2005) and can thus promote diverse and heterogeneous plant communities. The extent to which this creates good bee or butterfly habitat is unclear, however (Bendel et al., 2018; Buckles & Harmon‐Threatt, 2019; Moranz et al., 2012; Tonietto & Larkin, 2018).

## 6 CONCLUSIONS

The fact that bee and butterfly communities, with the exception of butterfly abundance, did not differ between sites managed with grazing or infrequent fire over a 13‐year period can be taken as an encouraging sign; the management practice that is most appropriate and practical in a given situation can be used without concern about harming invertebrate communities broadly, although some species appear to do better under one management practice. Burning, at least at sites managed with fire 1–3 times over 11 years, appears to support higher butterfly abundance, although this may be the result of untested variables and not the direct result of fire. Some species are more likely to be found in grazed sites and species composition differs with the number of years a site is managed. A variety of management strategies across sites is therefore important to support the entire suite of bee and butterfly species. Managers interested in promoting bee abundance and diversity might consider increasing forb frequency and targeting sites with sandier soils for acquisition, preservation, or future restoration.

## AUTHOR CONTRIBUTIONS

**Julia B. Leone:** Conceptualization (supporting); data curation (equal); formal analysis (lead); funding acquisition (supporting); investigation (lead); methodology (equal); project administration (supporting); resources (equal); software (lead); supervision (supporting); validation (lead); visualization (lead); writing – original draft (lead); writing – review and editing (lead). **Nora P. Pennarola:** Conceptualization (supporting); data curation (equal); formal analysis (lead); investigation (lead); methodology (equal); project administration (supporting); resources (equal); software (lead); supervision (supporting); validation (supporting); visualization (equal); writing – original draft (lead); writing – review and editing (supporting). **Jennifer L. Larson:** Conceptualization (supporting); data curation (equal); formal analysis (supporting); funding acquisition (supporting); investigation (equal); methodology (supporting); project administration (equal); resources (equal); supervision (supporting); validation (supporting); visualization (supporting); writing – original draft (supporting); writing – review and editing (supporting). **Karen Oberhauser:** Conceptualization (equal); formal analysis (supporting); funding acquisition (lead); investigation (equal); methodology (equal); project administration (equal); resources (equal); supervision (lead); validation (supporting); visualization (supporting); writing – original draft (supporting); writing – review and editing (equal). **Diane L. Larson:** Conceptualization (equal); data curation (supporting); formal analysis (supporting); funding acquisition (lead); investigation (equal); methodology (equal); project administration (equal); resources (equal); supervision (lead); validation (supporting); visualization (supporting); writing – original draft (supporting); writing – review and editing (equal).

## FUNDING INFORMATION

Funding for this project came from the Minnesota Environment and Natural Resources Trust Fund (ENRTF), M.L. 2015, Chp. 76, Sec. 2, Subd. 03o (all authors); Prairie Biotic Research, Inc. (JBL); National Science Foundation Graduate Research Fellowship (JBL); University of Minnesota Department of Entomology (NP); U.S. Geological Survey, Northern Prairie Wildlife Research Center (JLL, DLL). Any use of trade, firm, or product names is for descriptive purposes only and does not imply endorsement by the U.S. Government.

## CONFLICT OF INTEREST

All authors declare no competing interests.

## Data Availability

Data and metadata will be accessible through the Data Repository for University of Minnesota (DRUM): https://conservancy.umn.edu/handle/11299/166578.

## APPENDIX 1

**TABLE A1:** List of tallgrass prairie sites within the prairie parkland province in Minnesota, USA with management, local, and landscape details.^a^

Site ID	Year	Site area (ha)	Mgmt type	No. years managed	Time since fire	Stocking rate	Plant species richness	Forb frequency	*P. pratensis* and *B. inermis* frequency	Native graminoid frequency	Pct prairie	Prop sand
B‐1	2016	3.90	Burned	1	6	NA	32.85	5.10	0.83	0.92	9.63	0.26
	2017			1	7	NA	19.58	4.75	1.00	0.89		
B‐2	2016	1.42	Burned	1	6	NA	9.69	2.79	0.95	0.00	0.15	0.04
	2017			1	7	NA	14.13	3.67	1.00	0.17		
B‐3	2016	2.53	Burned	3	2	NA	19.87	7.63	0.13	1.00	25.77	0.15
	2017			3	3	NA	25.16	8.13	0.25	2.00		
B‐4	2016	4.10	Burned	1	7	NA	15.40	4.33	1.00	0.07	0.34	0.07
	2017			1	8	NA	12.21	3.60	1.00	0.13		
B‐5	2016	5.78	Burned	2	3	NA	33.87	9.06	1.00	3.71	6.79	0.07
	2017			2	4	NA	31.99	9.12	1.00	4.29		
B‐6	2016	23.60	Burned	1	5	NA	37.99	6.97	1.00	4.83	55.53	0.25
	2017			1	6	NA	30.82	6.49	1.00	4.90		
B‐7	2016	15.96	Burned	1	7	NA	32.30	4.78	1.00	2.46	4.43	0.26
	2017			1	8	NA	23.55	4.89	0.85	1.88		
B‐8	2016	37.21	Burned	2	2	NA	37.82	7.27	0.97	2.37	38.50	0.11
	2017			2	3	NA	25.03	6.67	0.90	1.20		
B‐9	2016	139.00	Burned	1	8	NA	26.42	6.43	0.63	3.40	38.27	0.08
	2017			1	9	NA	20.10	4.57	0.17	1.60		
B‐10	2016	39.30	Burned	2	4	NA	42.22	11.10	0.87	3.67	33.51	0.40
	2017			2	5	NA	38.71	10.10	0.97	6.56		
G‐1	2017	7.43	Grazed	3	NA	0.24	46.27	7.79	0.96	2.42	7.24	0.30
G‐2	2016	130.20	Grazed	12	NA	2.91	24.87	5.20	0.85	6.23	15.72	0.26
	2017			13	NA		31.15	5.93	0.87	2.89		
G‐3	2016	34.15	Grazed	12	NA	1.40	32.41	3.20	1.00	3.84	60.52	0.34
	2017			13	NA		24.18	3.23	0.97	4.07		
G‐4	2016	10.49	Grazed	2	NA	0.26	41.07	5.63	1.00	1.00	30.85	0.16
	2017			2	NA		24.81	5.77	0.93	1.13		
G‐5	2016	90.90	Grazed	12	NA	1.58	33.46	4.03	0.97	7.50	68.17	0.39
	2017			13	NA		26.61	4.27	0.97	5.79		
G‐6	2016	1.13	Grazed	4	NA	0.53	15.80	3.80	1.00	0.00	11.48	0.23
	2017			4	NA		9.00	1.60	1.00	0.00		
G‐7	2016	9.05	Grazed	5	NA	0.40	52.17	9.85	0.94	0.85	27.19	0.39
	2017			5	NA		31.59	7.69	1.00	2.19		
G‐8	2016	1.33	Grazed	3	NA	0.17	24.73	4.15	1.00	0.35	8.97	0.21
	2017			4	NA		16.22	4.35	0.95	0.35		
G‐9	2016	144.70	Grazed	12	NA	0.73	28.95	6.87	0.90	4.03	56.03	0.21
	2017			13	NA		30.18	6.60	0.87	2.36		
G‐10	2016	7.63	Grazed	12	NA	0.68	30.86	4.57	0.95	3.07	65.73	0.59
	2017			13	NA		27.86	5.67	1.00	4.86		

*Note*: These sites were exclusively either burned or grazed by cattle between 2005 and 2017 (10 burned, 10 grazed).

Abbreviation: NA, nonapplicable fields.

^a^ No. years managed indicates the number of years each site was managed between 2005 and 2017. Time since fire is the number of years since the site was last burned. Stocking rate is the average Animal Unit Month (AUM, or the number of cow‐calf units the site could support per month) at each grazed site for available years of stocking rate history, 2005–2015. “Mgmt” stands for Management, “Pct” stands for Percent (0%–100% prairie in surrounding 1.5 km site buffer), and “Prop” stands for Proportion (0–1 proportion sand within each site). Vegetation and soil variables were rounded to two decimal places.

## APPENDIX 2

Abundance and presence of butterfly and bee species observed in 2016 and 2017 at burned and grazed tallgrass prairie sites within the prairie parkland province in Minnesota, USA.

**TABLE A2:** Relative abundance and presence of butterfly species observed in 2016 and 2017 at burned and grazed tallgrass prairie sites within the prairie parkland province in Minnesota, USA.

Family	Genus	Species	Common name	Burned	Grazed
Hesperiidae	*Anatrytone*	*logan*	Delaware Skipper	2	2
Hesperiidae	*Ancyloxypha*	*numitor*	Least Skipper	27	32
Hesperiidae	*Atalopedes*	*campestris*	Sachem	1	2
Hesperiidae	*Hesperia*	*leonardus*	Leonard's Skipper^a^ ^,^ ^c^	0	1
Hesperiidae	*Poanes*	*viator*	Broad‐winged Skipper	0	1
Hesperiidae	*Polites*	*mystic*	Long Dash^a^ ^,^ ^c^	6	27
Hesperiidae	*Polites*	*peckius*	Peck's Skipper	M	13
Hesperiidae	*Polites*	*themistocles*	Tawny‐edged Skipper^a^ ^,^ ^c^	0	6
Hesperiidae	*Pyrgus*	*communis*	Common Checkered‐Skipper	4	0
Hesperiidae	*Thymelicus*	*lineola*	European Skipper		M
Lycaenidae	*Celastrina*	*neglecta*	Summer Azure	12	M
Lycaenidae	*Cupido*	*comyntas*	Eastern Tailed Blue	12	9
Lycaenidae	*Echinargus*	*isola*	Reakirt's Blue	1	0
Lycaenidae	*Glaucopsyche*	*lygdamus*	Silvery Blue	1	1
Lycaenidae	*Lycaena*	*hyllus*	Bronze Copper	M	1
Lycaenidae	*Lycaeides*	*melissa*	Melissa Blue^a^	1	M
Lycaenidae	*Satyrium*	*acadica*	Acadian Hairstreak	M	
Lycaenidae	*Satyrium*	*edwardsii*	Edwards' Hairstreak	M	
Nymphalidae	*Boloria*	*bellona*	Meadow Fritillary	29	32
Nymphalidae	*Boloria*	*selene*	Silver‐bordered Fritillary	1	1
Nymphalidae	*Cercyonis*	*pegala*	Common Wood Nymph^a^ ^,^ ^c^	73	39
Nymphalidae	*Coenonympha*	*tullia*	Prairie Ringlet^a^ ^,^ ^c^	0	3
Nymphalidae	*Danaus*	*plexippus*	Monarch	148	50
Nymphalidae	*Euptoieta*	*claudia*	Variegated Fritillary	1	6
Nymphalidae	*Junonia*	*coenia*	Common Buckeye	M	1
Nymphalidae	*Limenitis*	*archippus*	Viceroy	4	9
Nymphalidae	*Phyciodes*	*tharos or cocyta*	Pearl or Northern Crescent	113	29
Nymphalidae	*Satyrodes*	*eurydice*	Eyed brown^a^	10	3
Nymphalidae	*Speyeria*	*cybele*	Great Spangled Fritillary	53	2
Nymphalidae	*Speyeria*	*idalia*	Regal Fritillary^a^ ^,^ ^b^	31	12
Nymphalidae	*Vanessa*	*atalanta*	Red Admiral	9	6
Nymphalidae	*Vanessa*	*cardui*	Painted Lady	131	64
Nymphalidae	*Vanessa*	*virginiensis*	American Lady	2	M
Papilionidae	*Papilio*	*glaucus*	Eastern Tiger Swallowtail	M	M
Papilionidae	*Papilio*	*polyxenes*	Black Swallowtail	7	M
Pieridae	*Colias*	*philodice or eurytheme*	Clouded or Orange Sulfur	79	79
Pieridae	*Pieris*	*rapae*	Cabbage White	14	18

*Note*: M indicates species seen only during meandering walks. Abundance numbers represent butterflies observed during transect but not meandering walk surveys. *Phyciodes tharos* and *P. cocyta* were not distinguished during transect surveys for relative abundance, nor were *Colias philodice* and *C. eurytheme*.

^a^ Prairie‐associated butterfly species.

^b^ U. S. Fish and Wildlife Service Species of Concern.

^c^ Species included in the analysis of prairie‐associated grass feeders.

**TABLE A3:** Adjusted abundance and presence of bee species observed in 2016 and 2017 at burned and grazed tallgrass prairie sites within the prairie parkland province in Minnesota, USA.

Family	Genus	Species	Nesting habit	Burned	Grazed
Andrenidae	*Andrena*	*carlini*	Soil, excavator	1	0
Andrenidae	*Andrena*	*ceanothi*	Soil, excavator	1	7.63
Andrenidae	*Andrena*	*chromotricha* ^b^	Soil, excavator	0	1.53
Andrenidae	*Andrena*	*commoda* ^M^	Soil, excavator	M	
Andrenidae	*Andrena*	*cressonii* ^a^	Soil, excavator	35.8	24.87
Andrenidae	*Andrena*	*erythrogaster* ^a^	Soil, excavator	M	
Andrenidae	*Andrena*	*forbesii* ^b^	Soil, excavator	0	0.97
Andrenidae	*Andrena*	*helianthi* ^b^	Soil, excavator	0	0.97
Andrenidae	*Andrena*	*helianthiformis* ^a^	Soil, excavator	M	
Andrenidae	*Andrena*	*hirticincta* ^a^	Soil, excavator	M	
Andrenidae	*Andrena*	*milwaukeensis* ^b^	Soil, excavator	M	
Andrenidae	*Andrena*	*nivalis* ^a^	Soil, excavator	0	2
Andrenidae	*Andrena*	*placata* ^a^	Soil, excavator	1	0
Andrenidae	*Andrena*	*simplex* ^a^	Soil, excavator	M	
Andrenidae	*Andrena*	*thaspii*	Soil, excavator	0	1
Andrenidae	*Andrena*	*wilkella*	Soil, excavator	12.97	4
Andrenidae	*Andrena*	*ziziae*	Soil, excavator	0.9	2
Andrenidae	*Calliopsis*	*nebraskensis* ^a^	Soil, excavator	M	
Andrenidae	*Perdita*	*perpallida* ^b^	Soil, excavator	M	
Andrenidae	*Perdita*	*swenki*	Soil, excavator	0	10
Andrenidae	*Protandrena*	*bancrofti*	Soil, excavator	0	3
Apidae	*Anthophora*	*terminalis*	Aboveground	1	2
Apidae	*Anthophora*	*walshii* ^b^	Soil, excavator	M	
Apidae	*Apis*	*mellifera*	Aboveground	104.23	111.93
Apidae	*Bombus*	*auricomus*	Soil, nonexcavator	3	3
Apidae	*Bombus*	*bimaculatus*	Soil, nonexcavator	3	3
Apidae	*Bombus*	*borealis*	Soil, nonexcavator	8	0.67
Apidae	*Bombus*	*fervidus*	Soil, nonexcavator	17.8	14.93
Apidae	*Bombus*	*griseocollis*	Soil, nonexcavator	17.93	2
Apidae	*Bombus*	*impatiens*	Soil, nonexcavator	0	3
Apidae	*Bombus*	*pensylvanicus*	Soil, nonexcavator	1.8	10
Apidae	*Bombus*	*rufocinctus* ^a^	Soil, nonexcavator	M	
Apidae	*Bombus*	*ternarius*	Soil, nonexcavator	1	0.13
Apidae	*Bombus*	*terricola* ^a^	Soil, nonexcavator	M	
Apidae	*Bombus*	*vagans*	Soil, nonexcavator	3	2.83
Apidae	*Ceratina*	*dupla* ^b^	Aboveground	0	1.33
Apidae	*Ceratina*	*mikmaqi*	Aboveground	94.1	24.17
Apidae	*Eucera*	*hamata*	Soil, excavator	6	11
Apidae	*Melissodes*	*agilis*	Soil, excavator	7	8.67
Apidae	*Melissodes*	*bimaculatus*	Soil, excavator	0.93	19
Apidae	*Melissodes*	*communis* ^a^	Soil, excavator	1	0
Apidae	*Melissodes*	*denticulatus*	Soil, excavator	3	0
Apidae	*Melissodes*	*desponsus*	Soil, excavator	6	7.83
Apidae	*Melissodes*	*druriellus*	Soil, excavator	1	0.97
Apidae	*Melissodes*	*trinodis*	Soil, excavator	196.93	107.43
Apidae	*Nomada*	*articulata* ^a^	Soil, excavator	M	
Apidae	*Nomada*	“*near MR‐2*”^a^	–	M	
Apidae	*Svastra*	*obliqua* ^a^	Soil, excavator	M	
Apidae	*Triepeolus*	*donatus* ^b^	Soil, excavator	1	0.83
Apidae	*Xenoglossa*	*kansensis*	Soil, excavator	0	6
Colletidae	*Colletes*	*kincaidii*	Soil, excavator	4	0.53
Colletidae	*Colletes*	*robertsonii* ^a^	Soil, excavator	M	
Colletidae	*Colletes*	*simulans* ^a^	Soil, excavator	1	0
Colletidae	*Colletes*	*solidaginis* ^b^	Soil, excavator	0	1
Colletidae	*Colletes*	*susannae* ^a^	Soil, excavator	M	
Colletidae	*Hylaeus*	*affinis*	Aboveground	43.8	10
Colletidae	*Hylaeus*	*mesillae*	Aboveground	5.83	1.97
Colletidae	*Hylaeus*	*nelumbonis* ^b^	Aboveground	1	0
Halictidae	*Agapostemon*	*sericeus*	Soil, excavator	1.93	2.97
Halictidae	*Agapostemon*	*texanus*	Soil, excavator	18.67	285.63
Halictidae	*Agapostemon*	*virescens*	Soil, excavator	412.8	555.93
Halictidae	*Augochlorella*	*aurata*	Soil, excavator	834	355.33
Halictidae	*Augochloropsis*	*metallica*	Soil, excavator	3	1
Halictidae	*Halictus*	*confusus*	Soil, excavator	173.47	111.43
Halictidae	*Halictus*	*ligatus*	Soil, excavator	34.47	42.37
Halictidae	*Halictus*	*parallelus*	Soil, excavator	13.33	21.77
Halictidae	*Halictus*	*rubicundus*	Soil, excavator	14.93	6
Halictidae	*Lasioglossum*	*admirandum*	Soil, excavator	414.77	257.17
Halictidae	*Lasioglossum*	*albipenne*	Soil, excavator	956.53	562.97
Halictidae	*Lasioglossum*	*cattellae* ^a^	–	0.93	0
Halictidae	*Lasioglossum*	*cinctipes*	Soil, excavator	5.93	0
Halictidae	*Lasioglossum*	*coriaceum*	Soil, excavator	158.9	214.2
Halictidae	*Lasioglossum*	*cressonii*	Soil, excavator	9	40.23
Halictidae	*Lasioglossum*	*ellisiae*	–	5	53.6
Halictidae	*Lasioglossum*	*ephialtum*	Soil, excavator	56.33	55.27
Halictidae	*Lasioglossum*	*foxii* ^a^	Soil, excavator	1	0
Halictidae	*Lasioglossum*	*hitchensi*	Soil, excavator	3	2.67
Halictidae	*Lasioglossum*	*imitatum* ^a^	Soil, excavator	1	0
Halictidae	*Lasioglossum*	*laevissimum*	Soil, excavator	5.93	4
Halictidae	*Lasioglossum*	*leucocomum*	Soil, excavator	15.93	98.83
Halictidae	*Lasioglossum*	*leucozonium*	Soil, excavator	3	19
Halictidae	*Lasioglossum*	*lineatulum*	Soil, excavator	7.93	7.97
Halictidae	*Lasioglossum*	*michiganense*	Soil, excavator	0	3
Halictidae	*Lasioglossum*	*novasocotiae*	Soil, excavator	40.2	378.77
Halictidae	*Lasioglossum*	*paradmirandum*	Soil, excavator	1	1.8
Halictidae	*Lasioglossum*	*paraforbesii*	Soil, excavator	84.2	75.83
Halictidae	*Lasioglossum*	*pectorale*	Soil, excavator	2	7
Halictidae	*Lasioglossum*	*perpunctatum*	Soil, excavator	1.9	3
Halictidae	*Lasioglossum*	*pilosum* ^a^	Soil, excavator	0	3
Halictidae	*Lasioglossum*	*planatum*	Soil, excavator	1.93	4
Halictidae	*Lasioglossum*	*pruinosum*	Soil, excavator	743.53	1535.8
Halictidae	*Lasioglossum*	*semicaeruleum*	Soil, excavator	82.57	157.73
Halictidae	*Lasioglossum*	*subviridatum* ^a^	Aboveground	0	1
Halictidae	*Lasioglossum*	*tegulare*	Soil, excavator	2.83	57.5
Halictidae	*Lasioglossum*	*truncatum* ^a^	Soil, excavator	5	53.6
Halictidae	*Lasioglossum*	*versans*	Soil, excavator	1	0
Halictidae	*Lasioglossum*	*versatum*	Soil, excavator	0.93	0
Halictidae	*Lasioglossum*	*vierecki*	Soil, excavator	0	3
Halictidae	*Lasioglossum*	*viridatum*	Soil, excavator	5.8	0
Halictidae	*Lasioglossum*	*weemsi*	Soil, excavator	5.7	2
Halictidae	*Lasioglossum*	*zephyrum*	Soil, excavator	0	6.9
Halictidae	*Lasioglossum*	*zonulum*	Soil, excavator	9.97	6
Halictidae	*Nomia*	*universitatis* ^b^	Soil, excavator	M	
Halictidae	*Sphecodes*	*atlantis/cressonii* ^a^	Soil, excavator	0	1
Halictidae	*Sphecodes*	*davisii* ^b^	Soil, excavator	0	2
Halictidae	*Sphecodes*	*mandibularis* ^a^	Soil, excavator	1	0
Megachilidae	*Coelioxys*	*octodentata* ^a^	Aboveground	0	1
Megachilidae	*Coelioxys*	*rufitarsis* ^a^	Aboveground	1	0
Megachilidae	*Dianthidium*	*simile* ^b^	Soil, excavator	1	1
Megachilidae	*Heriades*	*carinata* ^b^	Aboveground	0	1
Megachilidae	*Heriades*	*leavitti*	Aboveground	M	
Megachilidae	*Hoplitis*	*Pilosifrons*	Aboveground	53.4	28
Megachilidae	*Megachile*	*brevis* ^b^	Aboveground	0.97	1
Megachilidae	*Megachile*	*latimanus*	Soil, excavator	10.97	5
Megachilidae	*Megachile*	*mendica* ^b^	Aboveground	1	0
Megachilidae	*Megachile*	*montivaga*	Aboveground	3	1
Megachilidae	*Megachile*	*relativa* ^b^	Aboveground	1	0
Megachilidae	*Osmia*	“*near collinsiae*”	–	3	0
Megachilidae	*Stelis*	*lateralis* ^b^	Aboveground	2	0

*Note*: Nesting habit designation is adapted from an in‐progress database from Bartomeus et al. (2013). M indicates species seen only during meandering walks. Adjusted abundance numbers represent bees collected during bee bowl but not meandering walk surveys.

^a^ Species represented by a single specimen (singleton).

^b^ Species represented by two specimens (doubleton).

## APPENDIX 3

**TABLE A4:** Random intercept variance values for final models.

	Random intercept variance in site	Random intercept variance in year nested within site
Total butterfly abundance	0.1037	0.1229
Butterfly resource‐user abundance	0.1225	0.1889
Non‐monarch butterfly abundance	0.07489	0.21137
Prairie‐associated grass‐feeding butterfly abundance	0.9207	0.1692
Bee abundance	2.668e−07	7.967e−01
Ground‐nesting bee abundance	1.746e−07	9.204e−01
Bee species richness	0.0166	0.1429

## APPENDIX 4

Univariate model results showing butterfly and bee responses to predictor variables.

**TABLE A5:** Univariate model results showing the response of total butterfly abundance to each predictor variable tested.

Model variable	Intercept				Variable				AIC	ΔAIC
	Est	SE	*z*	*p*	Est	SE	*z*	*p*		
All sites										
Null model	3.3061	0.1091	30.300	<2e−16	–	–	–	–	324.3	2.9
Management type (G)	3.5295	0.1346	26.216	<2e−16	−0.4522	0.1939	−2.332	.0197	321.4	0
Site area	3.3164	0.1360	24.386	<2e−16	−0.0003	0.0023	−0.127	.8990	326.3	4.9
Plant species richness	3.5898	0.2824	12.712	<2e−16	−0.0102	0.0094	−1.084	.2780	325.1	3.7
Forb frequency	3.2377	0.3012	10.749	<2e−16	0.0117	0.0480	0.244	.8080	326.2	4.8
Native graminoid frequency	3.3772	0.1678	20.123	<2e−16	−0.0281	0.0504	−0.558	.5770	326.0	4.6
Invasive thatch‐forming graminoid frequency	3.8258	0.4064	9.414	<2e−16	−0.5842	0.4430	−1.319	.1870	324.6	3.2
Percent prairie in buffer	3.3013	0.1769	18.662	<2e−16	0.0002	0.0049	0.034	.9720	326.3	4.9
Graze‐only sites										
Null model	3.0824	0.1142	26.99	<2e−16	–	–	–	–	149.7	0
Stocking rate	3.1612	0.1714	18.440	<2e−16	−0.0857	0.1414	−0.606	.544	151.3	1.6
Year managed	3.1131	0.2355	13.219	<2e−16	−0.0037	0.0250	−0.149	.881	151.7	2
Burn‐only sites										
Null model	3.5309	0.1579	22.36	<2e−16	–	–	–	–	170.6	0
Time since fire	3.4634	0.4058	8.535	<2e−16	0.0123	0.0680	0.181	.857	172.6	2
Year managed	3.5283	0.3863	9.135	<2e−16	0.0018	0.2349	0.008	.994	172.6	2

*Note*: ΔAIC indicates the relative differences between each univariate model and the “best‐ranked” (minAIC) model.

Abbreviation: AIC, Akaike information criterion.

**TABLE A6:** Univariate model results showing the response of butterfly resource‐user abundance to each predictor variable tested.

Model variable	Intercept				Variable				AIC	ΔAIC
	Est	SE	*z*	*p*	Est	SE	*z*	*p*		
All sites										
Null model	2.0339	0.1366	14.890	<2e−16	–	–	–	–	246.7	2.3
Management type (G)	2.2980	0.1654	13.890	<2e−16	−0.5353	0.2412	−2.220	.026	244.4	0
Site area	2.0919	0.1679	12.462	<2e−16	−0.0016	0.0028	−0.575	.565	248.4	4.0
Plant species richness	2.1534	0.3678	5.855	4.77e−09	−0.0043	0.0123	−0.349	.727	248.6	4.2
Forb frequency	1.6890	0.3751	4.503	6.7e−06	0.0590	0.0593	0.996	.319	247.7	3.3
Native graminoid frequency	2.0133	0.2107	9.555	<2e−16	0.0080	0.0627	0.128	.898	248.7	4.3
Invasive thatch‐forming graminoid frequency	2.3780	0.5231	4.546	5.47e−06	−0.3876	0.5719	−0.678	.498	248.3	3.9
Percent prairie in buffer	2.1071	0.2182	9.655	<2e−16	−0.0026	0.0060	−0.425	.671	248.6	4.2
Graze‐only sites										
Null model	1.7750	0.1518	11.690	<2e−16	–	–	–	–	109.3	0
Stocking rate	1.8274	0.2256	8.100	5.51e−16	−0.0584	0.1885	−0.310	.757	111.2	1.9
Year managed	1.8660	0.3019	6.180	6.41e−10	−0.0112	0.0324	−0.345	.730	111.2	1.9
Burn‐only sites										
Null model	2.2895	0.1996	11.470	<2e16	–	–	–	–	135.7	0
Time since fire	2.3649	0.5234	4.518	6.23e−06	−0.0137	0.0880	−0.156	.876	137.7	2
Year managed	2.1529	0.4816	4.471	7.8e−06	0.0911	0.2915	0.313	.755	137.6	1.9

*Note*: ΔAIC indicates the relative differences between each univariate model and the “best‐ranked” (minAIC) model.

Abbreviation: AIC, Akaike information criterion.

**TABLE A7:** Univariate model results showing the response of prairie‐associated grass‐feeding butterfly abundance to each predictor variable tested.

Model variable	Intercept				Variable				AIC	ΔAIC
	Est	SE	*z*	*p*	Est	SE	*z*	*p*		
All sites										
Null model	0.8813	0.2799	3.149	.0016	–	–	–	–	196.3	0.8
Management type (G)	0.8631	0.3854	2.239	.0251	0.0364	0.5251	0.069	.9448	198.3	2.8
Site area	0.6746	0.3512	1.921	.0547	0.0056	0.0053	1.059	.2895	197.1	1.6
Plant species richness	0.0425	0.5864	0.072	.9422	0.0302	0.0180	1.680	.0929	195.5	0
Forb frequency	0.7940	0.6808	1.166	.2430	0.0151	0.1069	0.141	.8880	198.3	2.8
Native graminoid frequency	0.5416	0.3869	1.400	.1620	0.1344	0.1004	1.338	.1810	196.5	1.0
Invasive thatch‐forming graminoid frequency	0.1168	1.0049	0.116	.9070	0.8528	1.0622	0.803	.4220	197.6	2.1
Percent prairie in buffer	0.4814	0.4571	1.053	.2920	0.0138	0.0117	1.178	.2390	196.9	1.4
Graze‐only sites										
Null model	0.9497	0.3293	2.884	.0039	–	–	–	–	99.7	0
Stocking rate	0.9836	0.4994	1.970	.0489	−0.0351	0.3937	−0.089	.9290	101.7	2
Year managed	0.7456	0.6876	1.084	.2780	0.0241	0.0695	0.346	.7290	101.6	1.9
Burn‐only sites										
Null model	0.8320	0.0037	226.2	<2e−16	–	–	–	–	100.3	1.3
Time since fire	1.3336	1.2579	1.060	.289	−0.0991	0.2319	−0.427	.669	102.1	2.1
Year managed	2.5665	0.9731	2.638	.0084	−1.1731	0.6659	−1.762	.0781	99.0	0

*Note*: ΔAIC indicates the relative differences between each univariate model and the “best‐ranked” (minAIC) model.

Abbreviation: AIC, Akaike information criterion.

**TABLE A8:** Univariate model results showing the response of butterfly species richness to each predictor variable tested.

Model variable	Intercept				Variable				AIC	ΔAIC
	Est	SE	*z*	*p*	Est	SE	*z*	*p*		
All sites										
Null model	3.0627	0.0773	39.600	<2e−16	–	–	–	–	296.3	0
Management type (G)	3.0657	0.1078	28.448	<2e−16	−0.0060	0.1539	−0.039	.969	298.3	2
Site area	3.1137	0.0956	32.582	<2e−16	−0.0014	0.0016	−0.875	.382	297.6	1.3
Plant species richness	3.0321	0.2354	12.878	<2e−16	0.0011	0.0080	0.138	.890	298.3	2
Forb frequency	3.0067	0.2216	13.560	<2e−16	0.0096	0.0356	0.270	.787	298.3	2
Native graminoid frequency	2.9564	0.1224	24.153	<2e−16	0.0418	0.0373	1.123	.261	297.1	0.8
Invasive thatch‐forming graminoid frequency	3.1099	0.3209	9.690	<2e−16	−0.0532	0.3513	−0.151	.880	298.3	2
Percent prairie in buffer	3.0099	0.1262	23.859	<2e−16	0.0018	0.0034	0.533	.594	298.0	1.7
Graze‐only sites										
Null model	3.0516	0.1246	24.490	<2e−16	–	–	–	–	142.9	0
Stocking rate	2.9624	0.1862	15.908	<2e−16	0.0977	0.1504	0.649	.516	144.5	1.6
Year managed	2.9651	0.2534	11.700	<2e−16	0.0106	0.0269	0.394	.694	144.8	1.9
Burn‐only sites										
Null model	3.0614	0.1142	26.800	<2e−16	–	–	–	–	156.8	0.8
Time since fire	3.4229	0.3035	11.277	<2e−16	−0.0658	0.0519	−1.268	.205	157.3	1.3
Year managed	2.6499	0.2616	10.129	<2e−16	0.2738	0.1567	1.747	.0806	156.0	0

*Note*: ΔAIC indicates the relative differences between each univariate model and the “best‐ranked” (minAIC) model.

Abbreviation: AIC, Akaike information criterion.

**TABLE A9:** Univariate model results showing the response of total bee abundance to each predictor variable tested.

Model variable	Intercept				Variable				AIC	ΔAIC
	Est	SE	*z*	*p*	*Est*	SE	*z*	*p*		
All sites										
Null model	5.3990	0.1540	35.060	<2e−16	–	–	–	–	534.7	3.5
Management type (G)	5.2403	0.2121	24.710	<2e−16	0.3250	0.3036	1.070	.284	535.6	4.4
Site area	5.5152	0.1895	29.103	<2e−16	−0.0032	0.0032	−1.028	.304	535.6	4.4
Proportion sand	4.7894	0.2901	16.511	<2e−16	2.5647	1.0595	2.421	.015	531.2	0
Forb frequency	5.5772	0.4445	12.547	<2e−16	−0.0306	0.0715	−0.428	.669	536.5	5.3
Invasive thatch‐forming graminoid frequency	4.7565	0.6417	7.412	1.24e−13	0.7231	0.7020	1.030	.303	535.6	4.4
Percent prairie in buffer	5.1537	0.2458	20.966	<2e−16	0.0085	0.0067	1.264	.206	535.1	3.9
Duration of bee bowl deployment	4.1283	1.7227	2.396	.0166	0.0003	0.0004	0.729	.466	535.5	4.3
Graze‐only sites										
Null model	5.5657	0.2107	26.420	<2e−16	–	–	–	–	273.0	2.9
Stocking rate	5.6267	0.3169	17.755	<2e−16	−0.0661	0.2570	−0.257	.797	270.1	1
Year managed	5.4136	0.4351	12.441	<2e−16	0.0462	0.399	0.445	.69	270.0	0
Burn‐only sites										
Null model	5.2400	0.2254	23.25	<2e−16	–	–	–	–	271.2	0
Time since fire	4.8697	0.6663	7.308	2.7e−13	0.0673	0.1137	0.592	.554	272.9	1.7
Year managed	5.4781	0.5456	10.040	<2e−16	−0.1588	0.3322	−0.478	.633	273.0	1.8

*Note*: ΔAIC indicates the relative differences between each univariate model and the “best‐ranked” (minAIC) model.

Abbreviation: AIC, Akaike information criterion.

**TABLE A10:** Univariate model results showing the response of ground‐nesting bee abundance to each predictor variable tested.

Model variable	Intercept				Variable				AIC	ΔAIC
	Est	SE	*z*	*p*	Est	SE	*z*	*p*		
All sites										
Null model	5.2762	0.1659	31.810	<2e−16	–	–	–	–	530.9	3.6
Management type (G)	5.0883	0.2278	22.338	<2e−16	0.3857	0.3261	1.183	.237	531.6	4.3
Site area	5.4434	0.2028	26.844	<2e−16	−0.0046	0.0034	−1.369	.171	531.1	3.8
Proportion sand	4.6120	0.3120	14.781	<2e−16	2.7980	1.1390	2.456	.014	527.3	0
Forb frequency	5.5077	0.4782	11.517	<2e−16	−0.0397	0.0769	−0.516	.606	532.7	5.4
Invasive thatch‐forming graminoid frequency	4.5866	0.6908	6.640	3.15e−11	0.7766	0.7556	1.028	.304	531.9	4.6
Percent prairie in buffer	5.0440	0.2658	18.975	<2e−16	0.0081	0.0073	1.108	.268	531.7	4.4
Graze‐only sites										
Null model	5.4749	0.2171	25.210	<2e−16	–	–	–	–	265.9	0
Stocking rate	5.5135	0.3269	16.864	<2e−16	−0.0418	0.2651	−0.158	.875	267.9	2.0
Year managed	5.3002	0.4480	11.832	<2e−16	0.0212	0.0475	0.445	.656	267.7	1.8
Burn‐only sites										
Null model	5.0870	0.2550	19.950	<2e−16	–	–	–	–	269.2	0
Time since fire	4.8474	0.7610	6.370	1.89e−10	0.0436	0.1301	0.335	.737	271.1	1.9
Year managed	5.2697	0.6211	8.485	<2e−16	−0.1217	0.3780	−0.322	.748	271.2	2

*Note*: ΔAIC indicates the relative differences between each univariate model and the “best‐ranked” (minAIC) model.

Abbreviation: AIC, Akaike information criterion.

**TABLE A11:** Univariate model results showing the response of total bee species richness to each predictor variable tested.

Model variable	Intercept				Variable				AIC	ΔAIC
	Est	SE	*z*	*p*	Est	SE	*z*	*p*		
All sites										
Null model	3.9305	0.0903	43.520	<2e−16	–	–	–	–	365.2	4.3
Management Type (G)	3.9260	0.1267	30.990	<2e−16	0.0090	0.1805	0.050	.9600	367.2	6.3
Site area	4.0070	0.1090	36.760	<2e−16	−0.0021	0.0018	−1.167	.2430	365.8	4.6
Proportion sand	3.8054	0.1814	20.979	<2e−16	0.5252	0.6621	0.793	.4280	366.5	5.7
Forb Frequency	3.3679	0.2174	15.491	<2e−16	0.0962	0.0347	2.774	.0055	360.9	0
Invasive thatch‐forming graminoid frequency	4.1790	0.3698	11.301	<2e−16	−0.2795	0.4037	−0.692	.4890	366.7	6.1
Percent prairie in buffer	3.9789	0.1459	27.281	<2e−16	−0.0017	0.0040	−0.421	.6730	367.0	6.1
Graze‐only sites										
Null model	3.9349	0.1054	37.32	<2e−16	–	–	–	–	179.2	2.9
Stocking rate	4.1534	0.1349	30.793	<2e−16	−0.2369	0.1111	−2.133	.0329	177.2	0.9
Year managed	4.2957	0.1838	23.367	<2e−16	−0.0437	0.0197	−2.216	.0267	176.9	0
Burn‐only sites										
Null model	3.9240	0.1464	26.810	<2e−16	–	–	–	–	190.5	0.3
Time since fire	4.4387	0.3543	12.530	<2e−16	−0.0935	0.0599	−1.560	.1190	190.3	0.1
Year managed	3.4589	0.3180	10.877	<2e−16	0.3101	0.1925	1.612	.1070	190.2	0

*Note*: ΔAIC indicates the relative differences between each univariate model and the “best‐ranked” (minAIC) model.

Abbreviation: AIC, Akaike information criterion.

**TABLE A12:** Univariate model results showing the response of ground‐nesting bee species richness to each predictor variable tested.

Model variable	Intercept				Variable				AIC	ΔAIC
	Est	SE	*z*	*p*	Est	SE	*z*	*p*		
All sites										
Null model	3.9194	0.1011	38.780	<2e−16	–	–	–	–	387.0	0
Management type (G)	3.8262	0.1400	27.334	<2e−16	0.1911	0.1997	0.957	.338	388.1	1.1
Site area	3.9625	0.1258	31.498	<2e−16	−0.0012	0.0021	−0.568	.570	388.7	1.7
Proportion sand	3.7193	0.2020	18.417	<2e−16	0.8413	0.7359	1.143	.253	387.7	0.7
Forb frequency	3.6608	0.2881	12.706	<2e−16	0.0443	0.0462	0.959	.337	388.1	1.1
Invasive thatch‐forming graminoid frequency	3.4177	0.4285	7.976	1.51e−15	0.5641	0.4678	1.206	.228	387.6	0.6
Percent prairie in buffer	3.9039	0.1644	23.750	<2e−16	0.0005	0.0045	0.120	.905	389.0	2
Graze‐only sites										
Null model	4.0202	0.1325	30.330	<2e−16	–	–	–	–	192.0	0
Stocking rate	4.2121	0.1901	22.160	<2e−16	−0.2080	0.1552	−1.340	.180	192.3	0.3
Year managed	4.2697	0.2656	16.077	<2e−16	−0.0302	0.0282	−1.069	.285	192.9	0.9
Burn‐only sites										
Null model	3.8226	0.1493	25.600	<2e−16	–	–	–	–	199.8	0
Time since fire	3.7518	0.4199	8.936	<2e−16	−0.0129	0.0712	0.181	.857	201.7	1.9
Year managed	3.8142	0.3669	10.396	<2e−16	0.0056	0.2242	0.025	.980	201.8	2.0

*Note*: ΔAIC indicates the relative differences between each univariate model and the “best‐ranked” (minAIC) model.

Abbreviation: AIC, Akaike information criterion.

## APPENDIX 5

Pearson and Kendall Correlations with ordination axes from nonmetric multidimensional scaling analysis of butterfly and bee species.

**TABLE A13:** Pearson and Kendall Correlations with ordination axes from nonmetric multidimensional scaling analysis of butterfly species.

Species	Axis 1			Axis 2			Axis 3		
	*r*	*r*‐sq	tau	*r*	*r*‐sq	tau	*r*	*r*‐sq	tau
Analog	.206	.042	.209	−.305	.093	−.369	−.001	.000	.010
Ancnum	.180	.033	.084	−.445	.198	−.387	−.453	.206	−.376
Atacam	−.402	.161	−.315	−.035	.001	.010	.045	.002	.112
Bolbel	.330	.109	.196	.276	.076	.035	−.076	.006	−.069
Bolsel	.212	.045	.145	.144	.021	.073	.418	.175	.218
Celneg	.257	.066	.070	.412	.170	.229	−.051	.003	−.209
Cerpeg	.509	.259	.269	.587	.345	.367	.380	.144	.455
Coetul	.134	.018	.117	−.346	.120	−.283	−.033	.001	−.050
Colspp	−.464	.215	−.434	.418	.174	.338	.245	.060	.198
Cupcom	−.399	.159	−.371	.138	.019	.146	−.373	.139	−.331
Danple	.621	.385	.505	.583	.339	.237	−.280	.078	−.290
Eupcla	−.119	.014	−.237	−.242	.058	−.059	−.221	.049	−.296
Glalyg	.188	.035	.097	.149	.022	.169	.249	.062	.145
Hemiso	−.089	.008	−.117	.292	.085	.250	−.150	.022	−.117
Hesleo	.134	.018	.117	−.346	.120	−.283	−.033	.001	−.050
Juncoe	.010	.000	−.050	−.211	.045	−.183	.583	.340	.316
Limarc	.099	.010	.173	.030	.001	−.121	.101	.010	.104
Lychyl	.024	.001	.017	.102	.010	.050	−.007	.000	.017
Lycmel	−.089	.008	−.117	.292	.085	.250	−.150	.022	−.117
Pappol	.191	.036	−.008	.494	.244	.416	−.098	.010	−.122
Physpp	.427	.182	.322	.683	.467	.420	−.135	.018	−.049
Pierap	−.267	.071	−.162	.203	.041	.114	−.181	.033	−.054
Poavia	.134	.018	.117	−.346	.120	−.283	−.033	.001	−.050
Polmys	−.509	.259	−.601	−.238	.057	.059	−.029	.001	−.088
Polpec	.136	.019	.107	−.292	.085	−.227	.547	.300	.370
Polthe	−.211	.044	−.083	−.416	.173	−.346	−.021	.000	.012
Pyrcom	.070	.005	.083	−.091	.008	−.083	−.218	.048	−.216
Sateur	.296	.087	.222	.456	.208	.237	.068	.005	.174
Specyb	.465	.216	.316	.584	.341	.276	.058	.003	.217
Speida	−.031	.001	−.235	.363	.132	.108	.266	.071	.323
Vanata	−.271	.074	−.334	.177	.031	.020	−.335	.112	−.321
Vancar	−.608	.369	−.525	.365	.133	.295	−.428	.183	−.219
Vanvir	−.089	.008	−.117	.292	.085	.250	−.150	.022	−.117

*Note*: Species are listed as first three letters of genus plus first three letters of species.

**TABLE A14:** Pearson and Kendall Correlations with ordination axes from nonmetric multidimensional scaling analysis of bee species.

Species	Axis 1			Axis 2			Axis 3		
	*r*	*r*‐sq	tau	*r*	*r*‐sq	tau	*r*	*r*‐sq	tau
Agaser	.206	.042	.240	−.085	.007	−.016	.098	.010	.032
Agatex	−.481	.232	−.374	.249	.062	.033	−.324	.105	−.110
Agavir	−.646	.417	−.453	.478	.228	.284	−.237	.056	−.168
Andbis	−.146	.021	−.150	.310	.096	.250	−.038	.001	−.050
Andcar	−.256	.065	−.347	.107	.012	.035	.203	.041	.364
Andcea	.215	.046	.083	−.057	.003	−.107	−.224	.050	−.131
Andcom	.014	.000	−.068	.352	.124	.239	.303	.092	.388
Andery	−.044	.002	−.083	−.233	.054	−.183	.181	.033	.250
Andfor	−.044	.002	−.083	−.233	.054	−.183	.181	.033	.250
Andmil	−.112	.013	−.117	−.366	.134	−.316	.006	.000	−.017
Andniv	.041	.002	.017	−.213	.045	−.150	−.183	.033	−.183
Andsim	−.012	.000	−.050	−.016	.000	−.050	.166	.028	.150
Andtha	.062	.004	−.099	.143	.021	.138	.174	.030	.217
Andwil	.009	.000	−.010	−.323	.104	−.269	.408	.167	.249
Andziz	.104	.011	.097	−.226	.051	−.218	−.405	.164	−.314
Antter	.065	.004	.091	−.167	.028	−.173	−.394	.155	−.335
Apimel	.263	.069	.142	.020	.000	−.016	−.414	.171	−.332
Augaur	−.476	.227	−.491	.358	.128	.248	.256	.065	.259
Augmet	−.238	.057	−.110	.089	.008	.050	.451	.204	.409
Bomaur	−.510	.260	−.355	.223	.050	.099	−.344	.118	−.217
Bombim	−.493	.243	−.317	.120	.014	.035	.042	.002	.106
Bombor	.367	.135	.243	−.269	.072	−.243	−.089	.008	−.121
Bomfer	.233	.054	.223	−.018	.000	−.006	−.185	.034	−.189
Bomgri	−.325	.106	−.096	.368	.136	.326	.115	.013	.045
Bomimp	−.190	.036	−.203	−.281	.079	−.179	.064	.004	.060
Bompen	−.318	.101	−.095	−.297	.088	−.190	.112	.013	.063
Bomter	.016	.000	−.107	−.345	.119	−.370	−.418	.175	−.179
Bomvag	.540	.292	.449	−.180	.032	−.128	−.476	.227	−.449
Cerdup	−.012	.000	−.050	−.016	.000	−.050	.166	.028	.150
Cermik	−.006	.000	.236	.210	.044	−.056	−.013	.000	.023
Coeoct	−.112	.013	−.117	−.366	.134	−.316	.006	.000	−.017
Coeruf	.098	.010	.117	.138	.019	.117	.109	.012	.050
Colkin	−.184	.034	−.250	.275	.076	.036	−.022	.001	.012
Colsim	−.146	.021	−.150	.310	.096	.250	−.038	.001	−.050
Colsol	.395	.156	.316	.075	.006	.050	−.310	.096	−.250
Diasim	−.541	.292	−.435	.314	.098	.290	−.150	.022	−.048
Eucham	−.568	.322	−.468	.015	.000	.104	−.058	.003	−.035
Halcon	−.081	.007	−.154	.218	.047	.122	−.058	.003	−.271
Hallig	−.581	.338	−.338	.325	.106	.172	−.049	.002	.039
Halpar	−.150	.023	−.137	.055	.003	−.041	−.059	.003	.014
Halrub	−.261	.068	.000	.277	.077	−.015	.047	.002	−.178
Hercar	−.012	.000	−.050	−.016	.000	−.050	.166	.028	.150
Hoppil	−.128	.016	−.023	.265	.070	−.035	−.038	.001	−.012
Hylaff	.153	.023	.028	.145	.021	.153	.050	.003	−.028
Hylmes	.569	.324	.444	−.300	.090	−.178	−.089	.008	−.074
Hylnel	.033	.001	−.017	.328	.107	.283	.213	.045	.283
Lasgro	−.395	.156	−.316	.248	.061	.216	−.387	.150	−.283
Lasadm	.286	.082	.381	.705	.497	.466	−.072	.005	−.095
Lasalb	−.372	.138	−.305	.555	.308	.411	.486	.236	.463
Lascat	.041	.002	.017	−.213	.045	−.150	−.183	.033	−.183
Lascin	−.030	.001	−.179	−.174	.030	.012	−.145	.021	.012
Lascor	−.102	.010	−.291	.067	.005	.000	.193	.037	.134
Lascre	.090	.008	.059	−.051	.003	−.085	−.044	.002	.085
Laseph	.633	.401	.434	−.067	.004	.022	−.354	.125	−.145
Lasfox	.041	.002	.017	−.213	.045	−.150	−.183	.033	−.183
Lashit	−.046	.002	−.044	.266	.071	.166	.068	.005	.026
Lasimi	.033	.001	−.017	.328	.107	.283	.213	.045	.283
Laslae	.304	.092	.237	−.233	.054	−.237	−.574	.330	−.474
Lasleu	−.428	.183	−.428	.268	.072	.123	−.379	.144	−.135
Lasleu	−.032	.001	.125	.423	.179	.110	−.559	.313	−.523
Laslin	.142	.020	.000	−.296	.088	−.223	−.534	.285	−.302
Lasmic	.088	.008	.083	−.001	.000	−.017	−.123	.015	−.117
Lasnov	.338	.114	.455	.423	.179	.195	−.328	.108	−.171
Laspar	−.144	.021	−.130	−.306	.093	−.269	−.242	.059	−.189
Laspar	−.272	.074	−.066	−.150	.023	−.066	−.414	.172	−.306
Laspec	−.281	.079	−.070	.239	.057	.149	−.331	.109	−.149
Lasper	−.149	.022	−.139	−.145	.021	−.171	−.235	.055	−.188
Laspil	−.116	.013	−.112	.268	.072	.213	−.348	.121	−.254
Laspla	.382	.146	.250	−.025	.001	−.060	−.366	.134	−.322
Laspru	−.689	.475	−.723	.335	.112	.202	−.287	.083	.128
Lassem	−.343	.118	−.206	−.291	.084	−.090	.283	.080	.259
Lassub	−.012	.000	−.050	−.016	.000	−.050	.166	.028	.150
Lasteg	−.409	.167	−.323	.230	.053	−.112	−.376	.141	.020
Lasteg	−.448	.201	−.329	.162	.026	−.317	−.416	.173	−.281
Lastin	−.146	.021	−.150	.310	.096	.250	−.038	.001	−.050
Lastru	.041	.002	.017	−.213	.045	−.150	−.183	.033	−.183
Lasver	.016	.000	−.044	−.152	.023	−.178	.086	.007	.030
Lasver	.353	.124	.374	.745	.555	.567	.075	.006	.118
Lasvie	−.395	.156	−.316	.248	.061	.216	−.387	.150	−.283
Lasvir	.130	.017	.183	.374	.140	.306	.132	.017	.079
Laswee	.138	.019	.217	−.190	.036	−.095	−.179	.032	−.078
Laszep	−.021	.000	−.079	−.297	.088	−.288	.127	.016	.148
Laszon	.216	.047	.276	−.062	.004	−.138	−.503	.253	−.395
Megbre	−.019	.000	−.036	.294	.086	.274	−.219	.048	−.203
Meglat	−.383	.147	−.324	.094	.009	.028	.107	.011	−.042
Megmen	−.349	.122	−.283	.184	.034	.183	.181	.033	.216
Megmon	.222	.049	.012	−.324	.105	−.274	−.113	.013	−.155
Megrel	.055	.003	.050	−.311	.097	−.283	−.434	.188	−.316
Melagi	.394	.155	.340	.093	.009	.007	−.049	.002	−.076
Melbim	−.106	.011	−.020	−.376	.142	−.276	.055	.003	.237
Melcom	.123	.015	.150	.040	.002	.017	.177	.031	.183
Melden	.137	.019	.155	−.105	.011	−.179	−.036	.001	−.083
Meldes	−.272	.074	−.140	.208	.043	.060	−.515	.265	−.432
Meldru	.345	.119	.274	.114	.013	.107	−.019	.000	−.060
Meltri	.457	.209	.342	.461	.212	.193	−.188	.035	−.182
Nomart	−.012	.000	−.050	−.016	.000	−.050	.166	.028	.150
Osmnea	−.146	.021	−.150	.310	.096	.250	−.038	.001	−.050
Perswe	−.407	.166	−.322	.207	.043	−.060	−.386	.149	−.227
Proban	−.381	.145	−.298	.120	.014	.036	−.271	.073	−.036
Sphatl	.088	.008	.083	−.001	.000	−.017	−.123	.015	−.117
Sphdav	−.395	.156	−.316	.248	.061	.216	−.387	.150	−.283
Sphman	.098	.010	.117	.138	.019	.117	.109	.012	.050
Stelat	−.146	.021	−.150	.310	.096	.250	−.038	.001	−.050
Tridon	−.007	.000	.036	.510	.260	.394	−.173	.030	−.179
Xenkan	−.163	.027	−.141	−.038	.001	.053	−.089	.008	−.158

*Note*: Species are listed as first three letters of genus plus first three letters of species.
