# Correction to Divergent responses of butterflies and bees to burning and grazing management in tallgrass prairies

**DOI:** 10.1002/ece3.11173

**Published:** 2024-03-17

Leone, J.B., Pennarola, N.P., Larson, J.L., Oberhauser, K. & Larson, D.L. (2022) Divergent responses of butterflies and bees to burning and grazing management in tallgrass prairies. *Ecology and Evolution*, **12**, e9532. https://doi.org/10.1002/ece3.9532.

There was an error in the soil texture report from the University of Minnesota Soil Testing and Research Analytical Laboratory. As a result, the *x* axis of Figure 4a is incorrect. The corrected graph is pictured here. Conclusions did not change as a result of this correction.

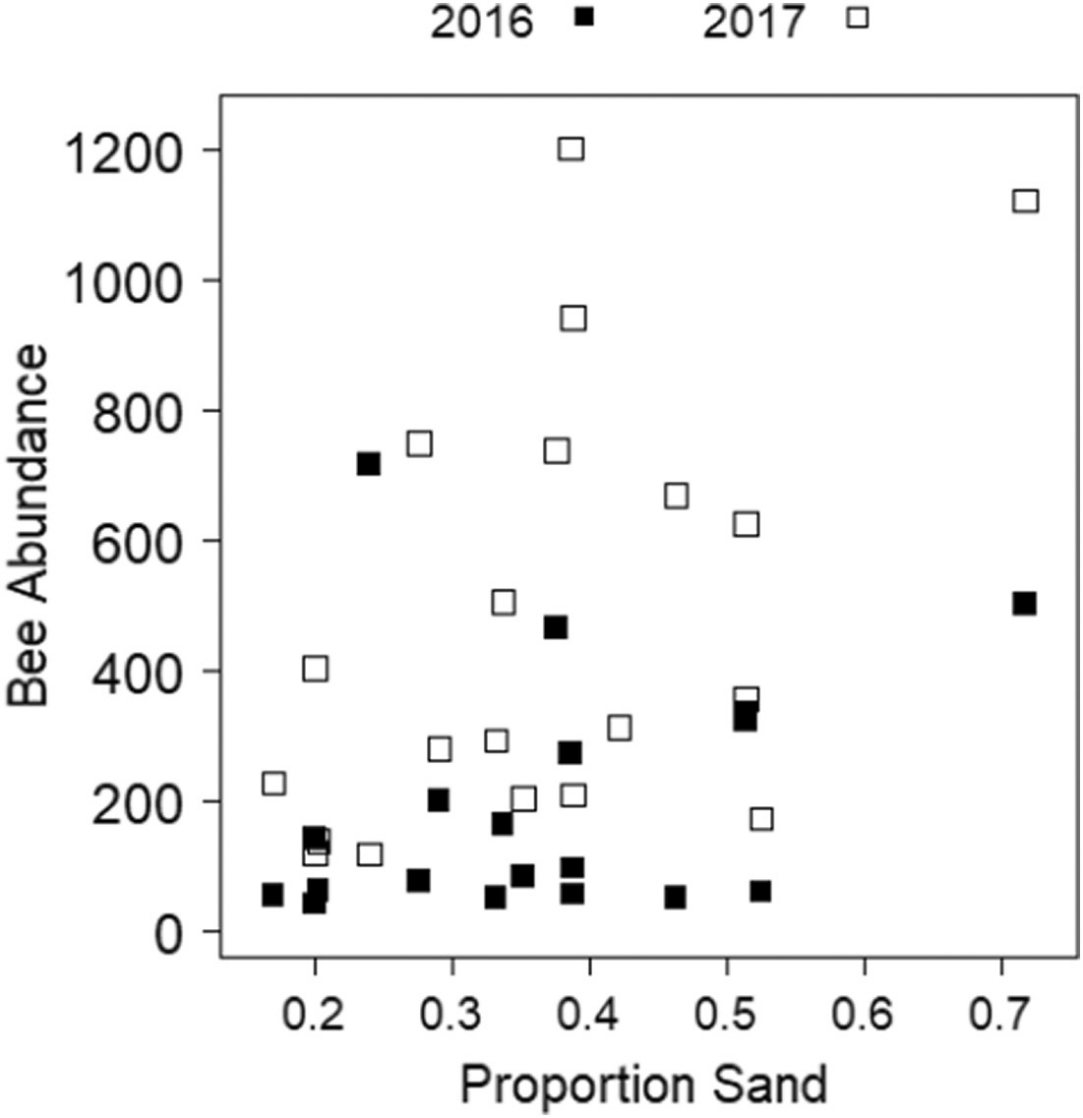

We apologize for this error.

